# Iron-regulated small RNA expression as *Neisseria gonorrhoeae* FA 1090 transitions into stationary phase growth

**DOI:** 10.1186/s12864-017-3684-8

**Published:** 2017-04-21

**Authors:** Lydgia A. Jackson, Michael Day, Jennie Allen, Edgar Scott, David W. Dyer

**Affiliations:** Department of Microbiology and Immunology, University of Oklahoma Health Sciences, 975 NE 10th Street, Oklahoma City, OK 73104 USA

**Keywords:** RNA-seq, Small RNA, Iron, Fur, *Neisseria gonorrhoeae*, NrrF, Riboswitch, Stationary phase, Transcriptome

## Abstract

**Background:**

For most pathogens, iron (Fe) homeostasis is crucial for maintenance within the host and the ability to cause disease. The primary transcriptional regulator that controls intracellular Fe levels is the Fur (ferric uptake regulator) protein, which exerts its action on transcription by binding to a promoter-proximal sequence termed the Fur box. Fur-regulated transcriptional responses are often fine-tuned at the post-transcriptional level through the action of small regulatory RNAs (sRNAs). Consequently, identifying sRNAs contributing to the control of Fe homeostasis is important for understanding the Fur-controlled bacterial Fe-response network.

**Results:**

In this study, we sequenced size-selected directional libraries representing sRNA samples from *Neisseria gonorrhoeae* strain FA 1090, and examined the Fe- and temporal regulation of these sRNAs. RNA-seq data for all time points identified a pool of at least 340 potential sRNAs. Differential analysis demonstrated that expression appeared to be regulated by Fe availability for at least fifteen of these sRNAs. Fourteen sRNAs were induced in high Fe conditions, consisting of both *cis* and *trans* sRNAs, some of which are predicted to control expression of a known virulence factor, and one SAM riboswitch. An additional putative *cis*-acting sRNA was repressed by Fe availability. In the pathogenic *Neisseria* species, one sRNA that contributes to Fe-regulated post-transcriptional control is the Fur-repressible sRNA NrrF. The expression of five Fe-induced sRNAs appeared to be at least partially controlled by NrrF, while the remainder was expressed independently of NrrF. The expression of the 14 Fe-induced sRNAs also exhibited temporal control, as their expression levels increased dramatically as the bacteria entered stationary phase.

**Conclusions:**

Here we report the temporal expression of Fe-regulated sRNAs in *N. gonorrhoeae* FA 1090 with several appearing to be controlled by the Fe-repressible sRNA NrrF. Temporal regulation of these sRNAs suggests a regulatory role in controlling functions necessary for survival, and may be important for phenotypes often associated with altered growth rates, such as biofilm formation or intracellular survival. Future functional studies will be needed to understand how these regulatory sRNAs contribute to gonococcal biology and pathogenesis.

**Electronic supplementary material:**

The online version of this article (doi:10.1186/s12864-017-3684-8) contains supplementary material, which is available to authorized users.

## Background

*Neisseria gonorrhoeae* colonizes the mucosal surfaces of the urogenital tract and causes gonorrhea, one of the most commonly reported sexually transmitted diseases. Due to the high incidence of infections and a global rise in multi-drug resistance strains, both the CDC and WHO have designated *N. gonorrhoeae* as a “super bug”, due to the pan-resistant nature of some strains in the gonococcal population [1]. We and others have reported the importance of the Fe-response regulon in infection and a wide range of cellular pathways including stress and oxidative responses [2–5]. The central transcriptional regulatory protein controlling the Fe regulon is the Fur protein which binds to conserved Fur box (FB) sequences in the promoters of Fe-responsive genes along with ferrous Fe^+2^ leading to transcriptional repression. As intracellular Fe stores are depleted, the Fur-Fe^+2^ complex dissociate and Fur is released from the promoter allowing transcription. Fur also has the capacity to induce gene transcription in response to Fe levels through both direct and indirect mechanisms (for a review see [6]). Indirect control of gene transcription is mediated through the action of secondary regulators including sRNAs [7]. In other bacteria, sRNAs control gene expression by post-transcriptional mechanisms during adaptation to environmental cues, including those provided by the host environment [8]. Dependent on their targets, sRNAs can be classified into two broad categories. *Cis*-acting sRNAs, varying in size from 100–7000 nucleotides (nt) are typically either riboswitches encoded in the 5′ UTR of the regulatory target or are co-transcribed antisense to the target gene [9]. Antisense sRNAs overlap all or part of the gene, or are located encoded on the 5′ or 3′ end of the protein-coding gene that they regulate. *Trans*-acting, typically 50–500 nt in length [10] most commonly target ribosomal binding sites near promoters, but may also exert regulatory control by binding to regulatory proteins and modifying their activity. Where *cis*-acting sRNAs generally target their cognate protein-coding RNA, *trans*-acting sRNAs bind targets on diverse mRNAs through imperfect base pair matches. In many gram negative organisms, these *trans*-acting sRNAs often require the RNA chaperone Hfq [11]. sRNAs often contain a Rho-independent terminator (RIT), an inverted repeat hairpin structure on the 3′ end that halts transcription of the sRNA.

Deep sequencing of Next Generation directional libraries and computational models to predict sRNAs have each dramatically increased sRNA discovery in many organisms [12]. In *N. gonorrhoeae*, whole transcriptome RNA-seq analysis of strain MS11 identified 253 potentially non-coding sRNAs of which 59 mapped to intergenic regions [13]. In another study, 232 potential sRNAs were identified from four different growth conditions (Fe-replete and deplete and co-culture in the presence or absence of endocervical cells) with seven confirmed by Northern analysis in gonococcal strain F62 [14]. More recently, NGS analysis of a *fur* mutant in F62 identified 13 small RNAs showing either increased or decreased expression compared to the wild type strain (WT) in the presence of Fe [15].

In the present study, we took a slightly different approach by taking advantage of deep-sequencing directional libraries to enrich for sRNA discovery across the growth curve allowing us to assess both temporal- and Fe regulation. We report 15 sRNAs regulated by Fe, of which 14 were induced in late log through stationary phase, with an additional sRNA expressed in low Fe conditions. Eight were putative *trans-*acting sRNAs with the remaining seven putative *cis*-acting sRNAs. We previously proposed that the Fe-repressible Neisserial sRNA NrrF functions to buffer the effects of Fur repression by fine-tuning mRNA turnover [16]. We were therefore interested in whether NrrF may have a similar effect on the expression of these Fe-regulated sRNAs. Transcriptional analysis of the 15 Fe-regulated small RNAs in an *nrrf* mutant strain identified 5 putative *trans*-acting small RNAs that appear to be regulated by NrrF.

## Results and discussion

### RNA-seq analysis of *N. gonorrhoeae* FA 1090 Fe regulated “sRNAome”

Size-selected directional libraries were sequenced from libraries prepared from the gonococcus grown in a defined medium under Fe-replete and –deplete growth conditions through stationary phase (1–5 h) (Additional file 1: Table S1). 150 bp single reads were aligned to the FA 1090 genome (NCBI Accession AE004969.1) and analyzed using RockHopper, a software designed for small RNA and transcriptome analysis of bacterial RNA-seq data [17] (Additional file 1: Table S1). Predicted sRNAs in the range >40 to <500 nt were examined for the presence of a promoter and RIT by extracting 150 bp 5′ and 3′ of the reads, using BPROM and ARNold, respectively [18–20].

**Fig. 1:**
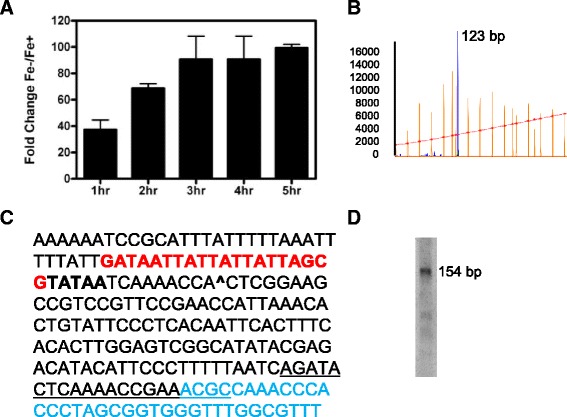
Transcriptional Analysis of Fe repressed sRNA NrrF. Iron regulation was confirmed with real time PCR transcriptional analysis of RNA samples isolated for RNA-seq from cells collected 1,2,3,4 and 5 h. **a** The TSS was resolved by primer extension using a FAM labeled primer yielding a 123 bp fragment as analyzed with Peak Scanner **b**) and a transcript length of 154 bp as determined by Northern blot analysis **d**). TSS for NrrF is indicated by an ^ in the NrrF sequence in **c**), with red denoting the FB sequence and bold type the −10 promoter region. Blue represents the RIT, with the unlined portion of the sequence the location of the FAM labeled primer used for the primer extension **c**)

Candidate sRNAs identified as potential coding sequences (GeneMark.HMM) [21] were subsequently omitted from the data set leaving a pool of 159 intergenic and 181 anti-sense sRNAs present in at least one time point during the growth curve (Additional file 2: Table S2). Expression of Fe-responsive genes and sRNAs such as NrrF in the gonococcus are directly regulated by Fur binding to FB consensus sequences near promoters [22], therefore; a computational approach was taken to predict FBs that may be associated with Fe-regulated sRNAs. Initially, using MEME analysis of a training set of FA 1090 sequences experimentally validated to bind Fur by FuRTA (Fur titration assay) and/or EMSA (Electrophoretic mobility shift assay) [4], an 18 bp direct repeat sequence was generated representing the Fur DNA-binding consensus sequence for FA 1090 (Additional file 3: Figure S1). HMM (hidden markov model) and PWM (position weight matrix) models were then built and each model was used to scan the entire FA 1090 genome. The predicted FB sequences extracted from both models were merged and imported into Artemis for visualization. The Fe-starvation status of cells used in these experiments was verified by assessing NrrF expression, which was highly repressed at all time points by real time PCR (Fig 1a). NrrF transcript size and transcriptional start site (TSS) are in agreement with previous studies (Fig 1b, c, and d) [23].

### Rfam database search of known regulatory small RNAs

**Fig. 2:**
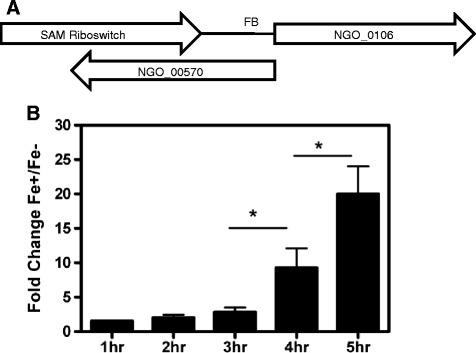
Fe Regulation of FA 1090 SAM Riboswitch. The SAM riboswitch is located in the 5′ UTR of the methionine adenosyltransferase gene NGO_0106 and anti-sense to hypothetical protein NGO_00570. FB indicates the presence of a Fur Box consensus sequence. **a** Real time PCR RNA samples results from cells harvested at 1,2,3,4 and 5 h showing induction of SAM riboswitch transcription in the presence of Fe beginning at late log and increasing into stationary phase. Each bar represents the mean fold change comparing Fe replete to Fe deplete growth conditions from three separate growth curves **b**). Asterisks indicate a significant increase in fold change values (*P* < 0.05)

Comparison of our RNA-seq data with the Rfam 12.0 database [24] revealed known regulatory RNAs including RNase P, tmRNA and a thiamine pyrophosphate (TPP) riboswitch, two tandem glycine riboswitches and one SAM riboswitch (Additional file 2: Table S2). Of the known regulatory RNAs, the SAM riboswitch was Fe-regulated (Fig 2b and data not shown). Riboswitches are a class of sRNAs that are often *cis*-acting regulatory loci originating in the 5′ UTR of the transcript they modulate by sensing metabolites and controlling genes involved in transport and synthesis of amino acids, metal ions, nucleotides and cofactors [25]. In FA 1090, the SAM riboswitch is located in the 5′ UTR of methionine adenosyltransferase gene (NGO_0106) and *cis* to a hypothetical protein NGO_00570 (Fig 2a) suggesting that this riboswitch also acts to regulate the expression of NGO_00570. The SAM riboswitch was not regulated by NrrF; as real time PCR transcriptional analysis showed no effect in the *nrrf* mutant (data not shown). The TSS and transcript length of 155 bp were determined by primer extension and Northern blot analysis (Additional file 4: Figure S2).

As the effector molecule S-adenomethionine (SAM) reaches a threshold concentration, binding to the riboswitch inhibits the downstream message through formation of a terminator. In the absence of SAM binding, an anti-terminator is formed and transcription of the downstream gene proceeds [26]. Presumably, the gonococcal SAM riboswitch functions in a similar manner by sensing SAM levels and disrupting methionine adenosyltransferase transcription as the ligand binds to the riboswitch. The SAM riboswitch in FA 1090 is induced in the presence of Fe with temporal transcriptional increases into stationary phase (Fig 2b). The methionine adenosyltransferase transcript is also directly regulated by Fur as evidenced by a predicted FB sequence in the promoter region (Additional file 4: Figure S2). This is consistent with the finding of a Fur ChIP-seq peak in the 5′ UTR of NGO_0106 in FA 1090 grown in Fe replete conditions (manuscript in preparation). Transcriptional profiles as determined by real time PCR confirm repression of the methionine adenosyltransferase transcript beginning at late log through stationary phase (data not shown). In this scenario, Fur binding to the FB sequence when Fe is present may exert a stronger regulatory effect on methionine adenosyltransferase transcription. If this is the case, studies in *Listeria monocytogenes* may suggest additional roles for the SAM riboswitch in the gonococcus. Loh et al. [27] demonstrated that terminated SAM riboswitch transcripts were able to function as a *trans*-acting sRNA regulating expression of a distal target through binding to the 5′ UTR of master virulence regulator *prfA*. Whether the gonococcal SAM riboswitch may function as a *trans*-acting sRNA potentially linking nutritional availability to the Fe regulon will require additional studies.

### sRNAs regulated temporally and by Fe-availability

We designated sRNAs with equivalent Fe regulation in WT and *nrrf* mutant strain LJ001, as Nrf (Neisseria sRNA Fe-regulated); see Table 1 for genetic designation and genomic location. Eight sRNAs were induced beginning in late log to stationary phase with one sRNA (NrfD) repressed in Fe-deplete conditions (Table 2). Four Fe-induced sRNAs previously have been reported in RNA-seq studies for *N. gonorrhoeae* strains MS11 and/or F62 [13, 14]. NrfA, NrfB, and NrfI (NgncR_002, NgnrC_021, and NgncR_239 respectively) were highly expressed in MS11 in the presence of Fe [13]. RNA-seq reads aligning to NrfA and NrfE were present in F62 strain however; these transcripts were not Fe regulated in the experimental conditions used in that study [14]. Expression of sRNAs; NrfC, NrfD, NrfF, NrfG and NrfH have not been reported in the gonococcus. Several of these novel Fe-regulated sRNAs merit further discussion.

**Table 1:** Genomic location of Fe regulated sRNAs

FA 1090	5′ ORF	sRNA Orientation	3′ ORF	Fe Regulation
NrfA	→NGO_0007	→ cis NGO_0010^a^	←NGO_0011	Induced
NrfB	←NGO_0184	→ cis NGO_0185^a^	←NGO_0186	Induced
NrfC	←NGO_0198	← trans	←NGO_0199	Induced
NrfD	→NGO_0274	→ cis NGO_0275	→NGO_0276	Repressed
NrfE	←NGO_0646	→ cis NGO_03400^a^	→NGO_0647	Induced
NrfF	→NGO_0773	← cis NGO_0775	→NGO_0777	Induced
NrfG	←NGO_1347	→ trans	←NGO_1349	Induced
NrfH	→NGO_1494	→ trans	←NGO_07895	Induced
NrfI	→NGO_2025	→ cis NGO_2026^a^	←NGO_2027	Induced

^a^RIT present

**Table 2:** Temporal expression of Fe regulated small RNAs

Fold Change in Expression (Fe+/Fe-)^a^					
FA 1090	1 h	2 h	3 h	4 h	5 h
NrfA	NC	NC	3.5 ± 1.00	13.2 ± 5.20	17.8 ± 7.20
NrfB	NC	NC	2.5 ± 1.10	4.9 ± 1.20	6.1 ± 2.40
NrfC	NC	NC	25.1 ± 0.02	283 ± 12.90	500.0 ± 80.00
NrfD^b^	−2.6 ± 0.40	−2.5 ± 0.90	−2.6 ± 1.10	−2.2 ± 0.05	−2.2 ± 0.40
NrfE	NC	NC	11.35 ± 1.50	32.4 ± 2.60	98.9 ± 31.00
NrfF	NC	NC	12.35 ± 2.50	24.4 ± 0.15	44.5 ± 0.55
NrfG	NC	NC	5.3 ± 1.30	8.6 ± 0.48	11.3 ± 1.00
NrfH	NC	NC	7.5 ± 0.95	13.6 ± 1.70	15.5 ± 2.30
NrfI	NC	NC	3.1 ± 1.30	7.6 ± 1.80	12.3 ± 0.60

^a^qRT_PCR fold change. Values reported are means for three experiments ± SEM

NC (no change) indicates a fold change of <2.0

^b^For fold change values <1, the reciprocal was taken and a minus sign added

Interestingly of the 15 sRNAs reported here NrfD was the only Fe repressed sRNA and did not exhibit temporal regulation with a modest 2-fold change in expression through all time points tested as determined by real time PCR (Table 2). The NrfD transcript is antisense to a 192 bp region of the protease domain in the *iga* gene (NGO_0275), (Additional file 4: Figure S2). IgA protease, a known virulence factor in pathogenic Neisseria is derived from a modular autotransporter protein directing the secretion of this serine protease to mucosal surfaces [28]. A genome-wide in silico analysis of FB locations in FA 1090 of the Fe-regulated sRNAs predicted a FB in the promoter region of NrfD. (Additional file 4: Figure S2). Although the regulatory role of intragenic FBs in controlling transcription is not fully understood, we have previously shown using EMSAs that Fur binds to two discrete intragenic FB sequences in *tbpA*, the gonococcal transferrin receptor protein [4]. Additionally, in a Fur ChIP-seq study, (manuscript in preparation) the intracellular FB sequence predicted near the promoter of NrfD bound Fur as detected by the presence of a peak overlapping that region in FA 1090, suggesting direct regulation of NrfD by Fur. Pathogenic Neisseria produce and secrete IgA protease in order to cleave IgA molecules, consequently interfering with the hosts’ innate mucosal immunity [29, 30]. In addition, IgA protease increases the degradation rate of host LAMP1 (lysosomal-associated membrane protein 1) a major membrane protein in lysosomal vacuoles, thereby promoting enhanced intracellular survival [31]. Although the mechanism whereby NrfD regulates the gonococcal IgA protease is not known; we speculate this potential *cis*-acting sRNA could play an important role in modulation of the host’s immune response at the mucosal surface and survival within host cells in response to Fe levels.

The NrfF transcript spans a 192 bp region antisense to the 3′ end of NGO_0775, harboring the proteolytic domain of Lon protease (Additional file 4: Figure S2) with transcription steadily increasing in the presence of Fe beginning at late log through stationary phase (Table 2). Bacteria adapt to environmental stresses by changing the overall protein profile through transcriptional control, but also through protein degradation by proteases. One important serine protease is the Lon protease, accounting for the majority of energy-dependent proteolytic activity in cells by targeting misfolded or damaged proteins for degradation during stress conditions as well as responding to DNA damage [32]. Lon protease and other ATP-dependent proteases have been shown to be involved in stationary phase adaptation through several regulatory pathways [33–35]. Given that Lon protease can exert a global effect, transcriptional regulation would be important to keep protein degradation in check. In fact, overexpression of Lon protease in *E. coli* leads to lethality [36]. Typically, *cis*-acting sRNAs modulate expression of their cognate RNA through binding and promotion of message degradation. Thus, a likely mechanism for NrfF action would be to regulate Lon protease expression post-transcriptionally in a similar manner. However, further studies are needed to clarify the role of NrfF in the Fe regulon and potential regulation of this important protease.

NrfG is oriented between membrane protein NGO_1347 and NGO_1349, a hypothetical protein (Table 1.) Of interest is a 106 bp Correia Repeat Enclosed Element (CREE) overlapping a region of the promoter of this Fe-induced sRNA (Additional file 4: Figure S2). CREE are 69–151 bp regions flanked by terminal repeats and a conserved core structure found only in *Neisseria* spp [37]. Insertion of CREE has been reported to inactivate genes [38]. Alternatively, CREE insertion adjacent to the 5′ end of a gene can create a promoter at either or both ends due to the presence of inverted repeats in the element [37]. A recent study investigated whether CREE are found near predicted sRNAs in gonococcal strain NCCP11945 [39]. RNA-seq data indicated the majority of CREE present in NCCP11945 were near or overlapping sRNA transcripts, implying CREE may be influencing sRNA expression [39]. Of the 123 CREE reported in FA 1090 [40], 36 or roughly 30% were located at or near the 5′ end or overlapping the length of the sRNA (Additional file 5: Table S3).

### NrrF regulated Fe dependent sRNAs

**Fig. 3:**
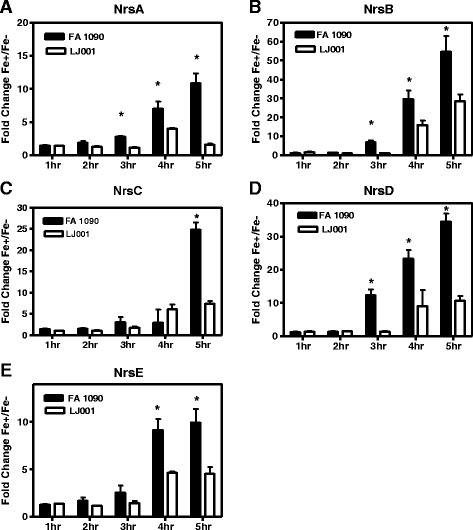
Temporal Expression of Fe Regulation in Nrs sRNAs. The levels of expression of Nrs sRNAs in FA 1090 compared to the mutant strain beginning at 3 h for NrsA, NrsB, and NrsD; 4 h or 5 h for NrsE, and NrsC. Expression continued to increase at subsequent time points according to real time PCR fold change results **a**-**e**). Values reported are means from three separate growth curves ± SEM at 1,2,3,4 and 5 h. Asterisks indicate a significant difference in fold change values of each Nrs sRNA in FA 1090 and in *nrrf* mutant strain (*P* < 0.05)

We identified five potential *trans*-acting sRNAs as being regulated by Fe availability and at least some component of Fe-regulation appeared to be mediated by NrrF (Fig 3). These sRNAs were additionally subjected to temporal regulation across the growth curve (Fig 3). We designated these Fe responsive sRNAs as Nrs (NrrF-regulated-small RNA); see Table 3 for name designation and genomic location. For all determinations of sRNA transcriptional levels as determined by real time PCR, the complemented NrrF strain LJ002 expressed levels similar to WT (data not shown). Three sRNAs have been reported in gonococcal strain MS11 [13]. NrsA (NgncR_036) and NrsD (NgncR_198) were found to be highly expressed in Fe-replete growth conditions with transcript lengths of 239 and 191 bps respectively [13]. The transcript lengths estimated from Northern blot analysis in FA 1090 were similar, 243 bp for NrsA and 193 bp for NrsD (Additional file 6: Figure S3). NrsB (NgncR_072) was also highly expressed in MS11 [13] with the 5′ end of this 404 bp transcript located antisense to a 98 bp region of the 3′ end of NGO_0686 in FA 1090. NrsB is a 145 bp transcript downstream of NGO_0686 with primer extension results corresponding to the TSS suggested by RockHopper analysis (Additional file 6 Figure S3). Novel transcripts NrsC and NrsE were 174 bp and 178 bp respectively as estimated by Northern blot analysis (Additional file 6: Figure S3).

**Table 3:** Genomic location of NrrF regulated Fe dependent small RNAs

FA 1090	5′ ORF	sRNA Orientation	3′ ORF	Fe Regulation
NrsA	→NGO_0331	← trans^a^	←NGO_0332	Induced
NrsB	→NGO_0686	→ trans	←NGO_0687	Induced
NrsC	←NGO_1221	← trans	←NGO_1222	Induced
NrsD	→NGO_1651	→ trans^a^	←NGO_1652	Induced
NrsE	→NGO_1713	→ trans	←NGO_1714	Induced

^a^RIT present

**Fig. 4:**
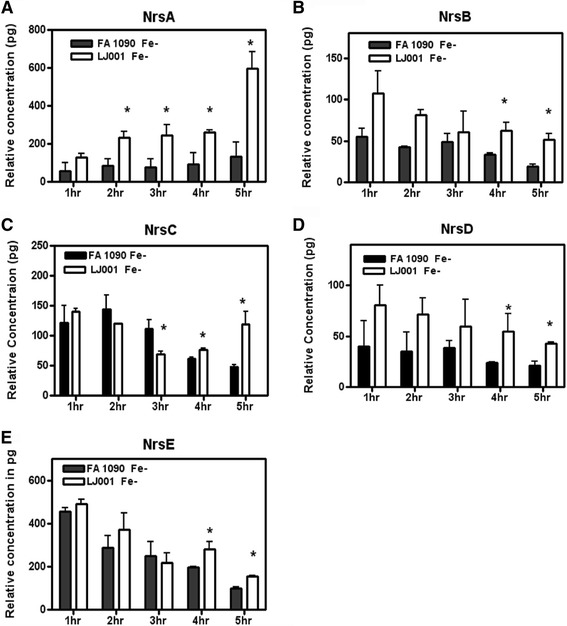
Relative Expression of NrrF Regulated Fe dependent Nrs sRNAs. The relative concentration of Nrs sRNAs was assessed by real time PCR in WT strain FA 1090 and *nrrf* mutant strain LJ001 in Fe restricted growth conditions. Values reported are means from three separate growth curves ± SEM at 1,2,3,4 and 5 h. Relative concentration significantly increased in the mutant strain compared to WT for Nrs sRNAs, with increased levels at late log and as early as 2 h **a**-**e**). *Difference in relative concentration between FA 1090 and *nrrf* mutant were significant at (*P* < 0.05)

Relative concentration of Nrs sRNA transcriptional levels was assessed in the WT and mutant strain when Fe was restricted. In the *nrrf* mutant there was a significant increase in transcription compared to WT in NrsB, NrsC, NrsD, and NrsE (Fig 4b, c, d and e) beginning at early stationary phase. Increased expression of NrsA in absence of NrrF was seen as early as 2 h (Fig 4a). These data indicate that reduced expression of Nrs transcripts was relieved during Fe restriction in the *nrrf* mutant. IntaRNA analysis of RNA-RNA interactions [41] using NrrF as the query and Nrs sRNAs to determine potential targets, suggested regions of complementary sequence between NrrF and each Nrs sRNA that were energetically favorable (Additional file 7: Figure S4). Using RNAfold [42] to predict the secondary structure of these regulatory sRNAs suggested that the base-pairing of NrrF with each Nrs sRNA was within an accessible region (Additional file 7 Figure S4).

Regulation by NrrF may not fully explain the temporal regulation when Fe is present and NrrF transcription levels are low. Progressive increases in the transcription of Nrs sRNAs began at late log and continued into stationary phase as evidenced by increasing fold change values as determined by real time PCR (Fig 3a-e). Moreover; the NrrF null mutant strain LJ001 exhibited a significant reduction in fold change as was evident at 3, 4, and 5 h for NrsA, NrsB, and NrsD (Fig 3a, b and d) and beginning at 4 h and 5 h, respectively for NrsC and NrsE (Fig 3c and e). NrrF regulation of these Fe-induced sRNAs through potential binding targets, or indirectly by additional regulators dependent on the availability of Fe in the organisms remains to be determined.

### Conservation of iron-regulated sRNA in *Neisseriaceae*

**Fig. 5:**
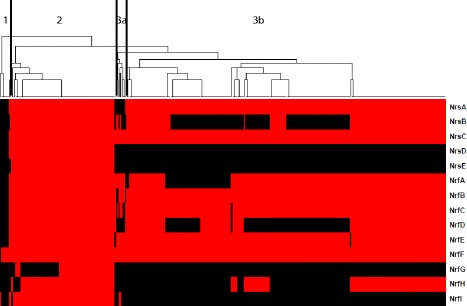
Heat map comparing Fe regulated sRNAs in FA 1090 to the *Neisseriaceae* family. This heat map is color-coded: Red representing the presence of the sRNA and black indicating the sRNA was absent. Hierarchical clustering analysis of the 14 Fe-regulated sRNA in 345 complete and draft genomes revealed four clusters. Cluster A was a small group of draft commensal genomes with limited homology to Nrf sRNAs. Commensals in cluster 3a showed a similar pattern of sRNA expression as cluster 3b containing the bulk of the meningococcal genomes. Gonococcal genomes were represented in cluster 2 with NrsD, NrsE and NrfG found only in *N. gonorrhoeae*. A larger version of this figure is provided in Additional file 10: Figure S5 to visualize *Neissericeae* genome names

The *Neisseriaceae* include commensal organisms commonly found on mammals as normal flora, in addition to the human pathogens *N. gonorrhoeae* and *Neisseria meningitidis*. To determine if the Fe regulated sRNAs reported in this study were shared by members of this family, we used blastn to query 451 genomes comprising complete and draft genome sequences available at NCBI (Additional file 8: Table S4). Sequences with a cut-off value of 85% length and 85% sequence identity for each Fe-regulated sRNA query were retained, resulting in 345 *Neisseria sp*. genomes with homology to one or more of the sRNAs (Additional file 8: Table S4). A heat map was constructed to cluster the Fe-regulated sRNAs in the commensal and pathogenic *Neisseria sp*. (Fig 5). Red indicates the presence of the sRNA while black denotes the absence. A larger version of this heat map that can more clearly visualize strain names is provided in Additional file 9: Figure S5. The 345 *Neisseria sp* genomes could be grouped into 4 clusters using hierarchical clustering (Fig 5; clusters 1, 2, 3a, 3b). Nrs sRNAs and the majority of the Nrf sRNAs were not found in cluster 1, which included *N. cinerea N. flavescens*, *N. macacae*, *N. mucosa*, and *N. sicca*. The *N. meningitidis* genomes in cluster 3b appear to have a heterogeneous cluster pattern for the NrrF regulated NrsA sRNA and several sRNAs in the Nrf sRNA group (Fig 5). *N. lactamica* and *N. polysaccharea* genomes present in cluster 3a were more similar to the sRNA content in *N. meningitidis* (Fig 5).

Compared to the diverse pattern seen in cluster 3, all Nrs and NrF sRNAs were present in *N. gonorrhoeae* complete reference genomes with a majority present in the draft genomes (Fig 5; cluster 2). Two NrrF regulated Nrs sRNAs, NrsD, NrsE, and Fe-regulated NrfG appear to be gonococcal specific (Fig 5). NrfD (antisense to known virulence factor IgA protease), NrfH, and NrsA are restricted to the pathogenic Neisseria; with NrfF, antisense to the protease subunit of the Lon protease, conserved in commensal and pathogenic *Neisseria sp.* (Fig 5; clusters 1, 2, 3a, and 3b).

## Conclusions

In this study, deep sequencing of directional sRNA libraries was utilized to assess sRNA expression relative to regulation by Fe and temporally across the growth curve; identifying 340 sRNAs. Of those candidate sRNAs; at least 15 including a SAM riboswitch were differentially expressed by the availability of Fe, with NrrF regulation of five. NrrF regulated Fe-dependent Nrs sRNAs NrsD and NrsE; and Fe-regulated NrfG were gonococcal specific (Fig 5).

**Fig. 6:**
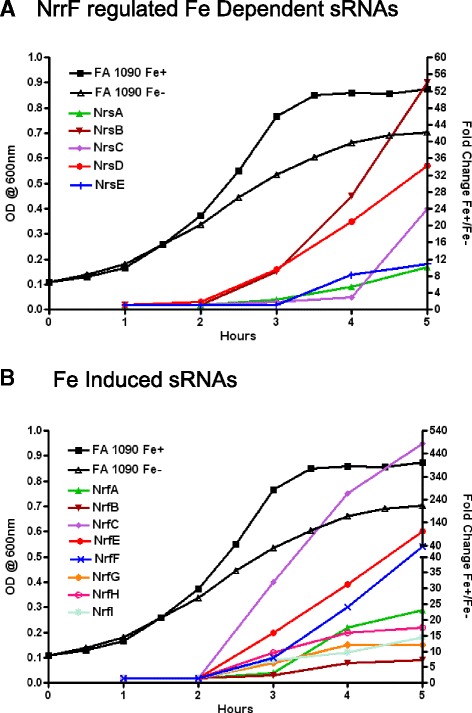
Temporal control of Fe regulated sRNAs. Real time PCR fold change results for all Fe-induced sRNAs were plotted against a representative example of an FA 1090 growth curve showing O.D. values across the 5 h time point to demonstrate the temporal increases in Nrs sRNA **a**) and Nrf sRNAs **b**). NrfD is not presented here as this sRNA was Fe-repressed and did not demonstrate temporal control

Strikingly, Nrs sRNAs (Fig 6a) and Nrf sRNAs (Fig 6b) were induced in the presence of Fe beginning at late log and increasing well into stationary phase with the exception of Fe-repressed sRNA, NrfD (Table 1). In fact, for several of these Fe-induced induced sRNAs it appears that transcription levels continue to increase to the 5 h time point (Fig 6). Growth in stationary phase is tightly regulated as organisms must adapt quickly to survive in stressful conditions [43]. In addition to known regulatory mechanisms governing this process [44] in *E. coli,* sRNAs have been implicated as regulators in stationary phase [45] as well as biofilm control [46]. Expression of sRNAs during stationary phase in other organisms has been reported [47–51]. For example, 140 sRNA were expressed as the organism entered early stationary phase in *Salmonella typhimurium* [49]. In *E. coli*, the majority of the 14 novel sRNAs identified were present in stationary phase with several reaching the highest expression going into late stationary phase [50]. Analysis of an extensive tiling array study in *N. meningitidis* using seven different growth conditions and stresses revealed that the majority of differentially expressed putative sRNAs were found in stationary phase [48]. How potential regulatory strategies for *N. gonorrhoeae* Fe-regulated sRNAs reported here may operate in the context of the Fe-regulon and physiology of the organism transitioning into stationary phase will be of great interest.

## Methods

### Bacterial strains and growth conditions

FA 1090 was grown from frozen stock cultures on GCB agar (Becton Dickinson) supplemented with 2% IsoVitaleX (Becton Dickinson) under 5% CO_2_ atmosphere at 37 °C. Fe-deplete (CDM-0) and Fe-replete medium (CDM-10), supplemented with 10 μm Fe (NO_3_)_3_ were prepared as previously reported [16, 52]. Briefly, organisms were inoculated to an OD_600_ = 0.100 from overnight growth on GCB agar plates. Cultures were grown to OD_600_ = 0.200 to deplete intracellular Fe pools. Half of the culture was transferred into a separate flask and CDM-0 was then added to both flasks to equal the original volume; Fe (NO_3_)_3_ was added to one of the flasks to a final concentration of 10 μM (CDM-10) [4]. The cultures were then allowed to grow to stationary phase in a 37 °C shaking incubator (225 rpm), and an aliquot of cells from Fe deplete and Fe replete conditions were collected at 1,2,3,4, and 5 h for RNA isolation.

### RNA isolation

Total RNA was isolated using a hot phenol method previously described [16] and pooled from three separate biological replicates for each time and growth condition as described above. RNA samples were DNase treated [1U RQ1 RNase-Free DNase per 1 μg RNA (Promega)] followed by using the TriReagent protocol for clean-up (Molecular Research Center, Inc.). RNA quantitation was done using the NanoDrop ND-100 (Thermo Scientific) and RNA integrity assessed using the Agilent RNA 6000 Nano Kit on the Agilent 2100 Bioanalyzer. Purified RNA samples were stored at −80 °C until further use.

### Transcript size selection

Total RNA was size selected for transcripts <500 bp following a protocol successfully used for sRNA sequencing in *Pseudomonas aeruginosa* [53]. Briefly, 50 μg DNAse-treated total RNA was separated by electrophoresis on a denaturing 10 M urea/10% polyacrylamide gel (Jule Biotechnologies Inc). The region of the polyacrylamide gel containing RNA of (50–500 nt) was excised, followed by centrifugation in Gel Breaker tubes (IST Engineering). The RNA was eluted overnight in 400 μl 0.4 M NaCL and filtered through a 0.5 μm filter tube (IST Engineering). RNA was extracted from the filtrate by addition of Phenol:Chloroform:Isoamyl alcohol (25:24:1, v/v) (Ambion). The aqueous phase was collected after centrifugation in a Phase-Lock Gel (5 Prime), followed by ethanol precipitation (0.02 volume 5 mg/ml glycogen (Ambion); 0.1 volume 3 M sodium acetate pH 5.5 (Ambion); 2.5 volumes cold 100% ethanol).

### Ribosomal RNA removal and TAP treatment

5 μg of size-selected RNA was depleted of 5S, 16S and 23S rRNA using the Ribo-Zero Magnetic Kit Gram Negative (Epicentre), followed by extraction in Phenol:Chloroform:Isoamyl alcohol and ethanol precipitation as described above. A portion of the rRNA depleted RNA samples were then treated with TAP (Epicentre) for 1 h @ 37 °C to convert 5′ phosphate-triphosphate to 5′-monophosphate, and then similarly purified and ethanol precipitated after the TAP reaction.

### Library production and sequencing

Directional libraries were prepared at Iowa State University DNA Facility http://www.dna.iastate.edu/nextgensequencing.html using the Illumina TruSeq Small RNA kit. All 10 directional libraries were bar-coded and 5 libraries were run per one lane of the Illumina MiSeq v3, to obtain 150 bp single reads. An average of 1,455,012 reads was generated from each library; this yielded an overall 62% alignment of high quality reads to the FA 1090 genome and an average 24% of those reads aligning to unannotated regions.

### Alignment and analysis of RNA-seq data

Reads were mapped to the *Neisseria gonorrhoeae* FA 1090 genome (NCBI GenBank Accession AE004969.1). Since the original FA 1090 annotation was over 10 years old, we re-annotated the FA 1090 genome sequence using the NCBI annotation pipeline on 6/22/2015. As an aid to the community, we developed an annotation conversion “look back” table to easily locate new locus tag numbers by querying old locus tag numbers using CD-Hit (Additional file 10: Table S5). RockHopper [17] was used for alignment and analysis of predicted sRNA.

### Prediction of DNA regulatory signals

Potential sRNA reads between 40 and 500 bp were retained and nucleotide sequence was extracted 150 bp 5′ and 3′ to each putative sRNA. These regions were screened for the presence of putative promoter motif using Promoter Prediction by PBROM (Softberry) [19] and a rho independent terminator with ARNold (Erpin and RNAmotif programs). For prediction of FB motifs in FA 1090, we used a training set of 23 sequences previously shown to bind Fur in vitro by EMSA and/or in vivo in a FurTA assay to generate a consensus FB sequence in MEME v 4.9.1. The FB consensus sequence generated in (WebLogo) [54] was 18 bp representing a 7-1-7 inverted repeat (Additional file 3: Figure S1). In parallel, we built a hidden markov model (HMM) using hmmer v 1.8.5 and position weight matrix (PWM) model (MEME v 4.9.1). Both models were used to scan the FA 1090 genome. Leave-one-out cross validation (LOOCV) [55] was used to determine an empirical *p*-value cut-off of 0.008 for the HMM model and *p*-value cut-off of 0.00103 for the PWM model.

### sRNA secondary structure and NrrF target predictions

RNAfold [42] was used to generate the secondary structure for NrrF and Nrs sRNAs on the ViennaRNA Web Server http://rna.tbi.univie.ac.at/. IntaRNA [41], a tool for prediction of RNA-RNA interactions was used to determine possible targets of NrrF in the Nrs RNAs. http://rna.informatik.uni-freiburg.de/IntaRNA/Input.jsp.

### Rfam homology search to known regulatory ncRNAs

Homology to known regulatory ncRNAs was done by blasting the pool of candidate sRNAs to the Rfam 12.0 release database (http://rfam.xfam.org) [24] using the default scoring. Hits with an e-value less than 1 were reported.

### *Neisseriaceae* homology search and heat map

Complete and draft genomes of 451 members of the *Neisseriaceae* family including both commensal and pathogenic organisms used in this study are listed in Additional file 8: Table S4. Blastn (NCBI Nucleotide v 2.2.28) of the 451 genomes was performed using the default *p* = 0.05, cut-off of ≥85% of sRNA query length and ≥85% sequence identity to Nrs and Nrf Fe regulated sRNAs. The blastn results were tabulated in a table of all genomes present in the blastn report vs. the blastn results for each of the 14 sRNAs. Scores of 0 or 1 were assigned to the following states; 0 for genomes not found for that specific sRNA and 1 for a hit indicating the presence of that sRNA. In-house scripts in R version 3.2.3 were written to reduce the number of draft genome duplicate contigs for presentation in a table and heat map and to bundle all contigs of same strain together into a single genome name. Clustering analysis for this heat map was generated using the default hclust function in the gplots package of R.

### qRTPCR

To estimate sRNA levels, quantitative real time PCR (qRT_PCR) was performed as previously described [16]. Briefly, cDNA was generated from DNAse-treated total RNA using the High Capacity cDNA Archive Kit using random primers and including a negative control reaction lacking the reverse transcriptase enzyme (RT; Applied Biosystems). RT using gene specific primers (GSP) were set up as per the High Capacity cDNA Archive Kit with a few modifications. The reverse real time primers were used as the GSP for RT of antisense sRNAs. All GSP were at 0.2 pM final concentration with a 20 min annealing temperature at the respective primer Tm, replacing the 10 min room temperature annealing temperature as for RT using random primers. Amplification of all cDNA was performed on an ABI 7500 Fast Real-Time PCR system (Applied Biosystems) using SYBR green master mix (Applied Biosystems). The relative expression was calculated by the comparative threshold cycle (2^-ΔΔCT^) method, with fold changes calculated as a ratio of the qRT-PCR measurements of sRNA from the Fe-deplete/Fe-replete growth conditions. The relative concentration was reported in pg and quantified using the standard-curve method (user bulletin no. 2; Applied Biosystems). Real-time reactions were carried out in duplicate with *porA* as the endogenous reference. Primers and annealing temperatures are listed in Additional file 11: Table S6.

### Fluorescent Primer Extension (FPE)

TSS was estimated using primer extensions employing fluorescent primers [56]. Reactions were performed by addition of FAM-labeled primer (0.01 μM final concentration) to 20–30 μg of DNase treated total RNA (20 μl total reaction volume), heated for 5 min @ 70 ° C and placed on ice for 10 min. Samples were then incubated for 20 min at the specified primer annealing temperature and cooled to room temperature for 15 min. The reverse transcription reaction was performed as follows: annealed RNA/primer mixture, 400 U RT (SuperScript III Invitrogen), (1 mM dNTP and 0.01 M DTT final concentration) in a total reaction volume of 40 μl. The reaction was incubated for 2 h at 42 ° C followed by sodium acetate/ethanol precipitation. Fragment analysis was done on the ABI 3730 DNA analyzer using GeneScan 600-LIZ ladder at the Laboratory of Molecular Biology and Cytometry Research http://research.ouhsc.edu/CoreFacilities.aspx. A standard curve was generated in Peak Scanner (Applied Biosystems) software to calculate the size and intensity of the FAM labeled cDNA products. Primers and annealing temperatures are listed in Additional file 11: Table S6.

### Northern blot analysis

Northern blots were performed by addition of 20ug of DNAse treated 5 h Fe replete RNA with an equal amount of 2× Gel loading buffer II (Life Technologies P/N: AM8547), denatured for 10 min at 95 °C, and loaded onto a denaturing 15% TBE-Urea polyacrylamide gel (Invitrogen). Gels were run for 3 h at 150 V and then transferred onto Hybond-N+ membrane (GE Healthcare) by wet blotting at 12 V for 2 h. RNA was then UV cross-linked twice to the membrane at 120 mJ/cm^2^ (Fisher Scientific). Probes were generated by labeling the 3′ end of the 5′ FAM-labeled oligonucleotides used for primer extensions (Additional file 11: Table S6) according to the manufacturer’s protocol (Roche cat. number 03353575910). Hybridization was performed overnight at 10 °C below the FAM-labeled oligonucleotide Tm in a ProBlot hybridization oven (Labnet International). Probe detection was performed using the DIG luminescent detection kit (Roche) according to the manufacturer’s protocol. Briefly, membranes were blocked in blocking buffer for 30 min at 25 °C, then incubated in antibody solution (anti-DIG antibody diluted in blocking buffer; 1:10,000) for 30 min at 25 °C, then washed twice in 1× washing buffer (0.1 M maleic acid, 0.15 M NaCl at pH 7.5, 0.3% Tween 20 [v/v]) for 15 min at 25 °C. Membranes were then equilibrated in detection buffer (0.1 M Tris, 0.1 M NaCl, pH9.5); incubated in 0.25 mM CDP-star at room temperature for 5 min and then exposed on the Kodak Gel Logic 1500.

### Illumina RNA-seq data accession number

RNA_seq data are deposited at Gene Expression Omnibus (GEO) https://www.ncbi/nih.gov/geo under BioProject PRJNA356970.

### Statistical analysis

Results were expressed as the standard error of the mean (SEM). Paired *t* test was used for all comparisons. A *P* value of <0.05 was considered significant.

## Additional files

Additional file 1: Table S1. Complete list of all reads in FA 1090 from Rockhopper analysis at 1, 2, 3, 4, and 5 hrs in Fe minus and Fe plus growth conditions. Representative growth curves for FA 1090, LJ001 *nrrf* mutant strain and NrrF complemented strain LJ002. (XLSX 302 kb)

Additional file 2: Table S2. List of the 340 candidate sRNAs reported in FA 1090 at 1, 2, 3, 4, and 5 h in Fe minus and Fe plus growth conditions. (XLSX 90 kb)

Additional file 3: Figure S1. Fur box consensus sequence representing the Fur DNA-binding region in FA 1090 generated by Weblogo. (DOCX 100 kb)

Additional file 4: Figure S2. Fe regulated Nrf sRNAs; primer extensions and Northern blot analysis. (DOCX 1051 kb)

Additional file 5: Table S3. CREE associated with sRNA reads at 1, 2, 3, 4, and 5 h in Fe minus and Fe plus growth conditions. (XLSX 22 kb)

Additional file 6: Figure S3. Fe dependent NrrF regulated Nrs sRNAs; primer extensions and Northern blot analysis. (DOCX 611 kb)

Additional file 7: Figure S4. NrrF regulated Fe dependent Nrs sRNAs; prediction of secondary structure using RNAfold; IntaRNA predictions of potential RNA-RNA interactions between NrrF and the Nrs sRNAs. (DOCX 811 kb)

Additional file 8: Table S4. Lists of 451 Neisseriaceae complete and draft genomes; blastn results of the homology search in 345 *Neisseria sp.* for Nrs and Nrf sRNAs; list of the *Neisseria sp.* genomes used for cluster analysis. (XLSX 353 kb)

Additional file 9: Figure S5. Heat map cluster analysis of Fe regulated sRNA in the 345 *Neisseria sp.* genomes. Red indicates the presence of the sRNA and black designates that the sRNA is absent from that genome. (PDF 32 kb)

Additional file 10: Table S5. Look back table for FA 1090 genome annotation generated by CD-Hit. This annotation conversion table allows query of old locus tags to locate new locus tags. (XLSX 287 kb)

Additional file 11: Table S6. List of the strains and the primers used in this study for real time PCR, primer extension, and Northern blot. (DOCX 18 kb)

## Abbreviations

FB: Fur box
Fe: Iron
Fur: Ferric uptake regulator
mRNA: Messenger RNA
NGS: Next generation sequencing
nt: Nucleotides
ORF: Open reading frame
RIT: Rho independent terminator
sRNA: Small RNA
TSS: Transcriptional start site
WT: Wild type
