# Supplementary material for: Iron-regulated small RNA expression as Neisseria gonorrhoeae FA 1090 transitions into stationary phase growth
Source: BMC Genomics. 2017 Apr 21;18:317. doi: 10.1186/s12864-017-3684-8 (PMC5399841; doi:10.1186/s12864-017-3684-8)
Supplement: Supplementary file 3 — Fur box consensus sequence representing the Fur DNA-binding region in FA 1090 generated by Weblogo. (DOCX 100 kb) [file 12864_2017_3684_MOESM3_ESM.docx]

Additional_file_3_Figure_S1_FA_1090_FB_consensus_sequence_MEME_plasmid _training_sequences

A.

The FB logo representing the Fur DNA-binding region was generated using MEME analysis of FA 1090 training set of 23 sequences previously shown to bind Fur in vitro by EMAS and/or in vivo in a FurTA assay (ref). The FB sequence is 18 bp, representing a 7-1-7 inverted repeat. The bit score, or overall height represents sequence conservation at a given position, and the height of each residue within each stack represents the frequency of the residue.

B. FA 1090 plasmid sequence training set for MEME

>FA_1090_pDFB10

CAGGCAGTTCGACCTCATTGTGATGGACCCGCCCAGTTTTTCCAACAGCAAGAAAATGCC

CGGCATCCTCGACATCCAGCGCGACCATCAAAAACTCATAGACGGCGCGGTGAACCTGCT

CGCTTCAGACGGCATTTTGTATTTTTCCAACAACCTGCGCAGCTTCGTTTTGGACGATTC

GGTATCGGAACAATACGCCGTGAAAGACATTTCCAAACAATCCGTTCCCGAAGATTTCCG

CAACAAAAAAATCCACCGGTGTTGGGAAATCCGCCACAAATCTTAAGGGTGCGAAAAAGC

CGCCGGAATCTTCCGGCGGCTTTTCGATTGCGCTTTATCAGGCGATGGAGGCCTCGACAA

AGGCGGTCAGCTGACCTTTTGCCAATGCGCCGACTTTGGTGGCGACGACTTCGCCGTTTT

TGAACACCATCAGGGTCGGAATGCCGCGCACGCCGAAACGGGACGGGGTGGCTTCGTTGT

CGTCGATGTTGATTTTGACTACTTTCAGACGGCCTTCAAATTCGGCGGCAATGTCGTCCA

AAATCGGGGCAATCATTTTGCAGGGGCCGCACCACGGAGCCCAAAAGTCCAGCAGGACGG

GGAGGTCGGATTTCAAAACATCTTGCTCGAAGGCGGCATCGCCGGTGTGTACGATTAATT

CGCTGCTCATTATTTTTCCTTTTCGGTTGGGATTCGCACGATAGCGTGCAGGATAGGGCT

TGGGCATAAAAATTTCAAGTGGGCGGGCGGGATAATAGTTTTTCTGTACCGCCCCAATCG

CCTTTGCAAATTCTAAAAAACGGTTCGGACAGTATTTTATTTTTGAAAATTACTTTTATT

ATCAAAGTATAAGGAATGTTTTTGAGGCTGTACAGAAACGCGCGGGCGGTGTATGCTCCG

TTTAAATAGATTCTTAAAGTATGAATGTTCAAAACCAAACCCGAAACCCTATAAAAGGAA

CACTGCAATGACTGATTTTTCCGTTTGGGAAGCTGCTCCCTTTGGCGCAACCGTTGATCA

TATCCTGCAACGCTACCACAATGTCCACCGCGCACAATTTGAAGAATTGGTGCCGCTGGC

GCAGAAAGTGGCCCAAGTCCATGCCGATACCTTTCCGGCGGAAATTGCCGGGCTGCTTGC

CGATATGCGGGACGAGCTGCTGATGCATA

>FA_1090_pDFB11

AATGCCGAGGAAGATAAAAAGCTGACTGAATTGGTCGCTTCCCGCAACCAAGCCGAAGCC

CTGATTCACTCCGTGAAAAAATCTTTGGCGGACTACGGCGACAAACTCGACGCTGCCGAG

AAAGAAAAAATCGAAGCCGCGCTGAAAGAAGCCGAAGAAGCCGTGAAAGGCGACGACAAA

ACCGCCATCGATGCCAAAGCCGAAGCACTGGGTACAGCCAGCCAAAAACTGGGCGAAATG

GTTTACGCGCAAGCGCAAGCCGAAGCCCAAGCCGGCGAGGGCGCACAAGCCAATGCTTCT

GCAAAGAAAGACGATGATGTCGTAGATGCCGACTTTGAAGAAGTAAAAGACGACAAAAAA

TAATTGATGCCGTCTGAAAAAAACGCGAACCATTCGGTTCGCGTTTTTTTCAATTGAGAT

AAAAGACAATAGCATGACAGAGATTCCAAATTCATCTACCGTGATATTCCCAAGCCCAAG

TTCCAACTGTTGCATCGGTTATCTGGAAATTTTTCATATCATTTTCTTATTTACTTAAAT

TTTTTAATAAGATAATAAATAATAATTATTATCATGAGCTAAGAAATGAAACTAAATACT

CTCACATGGGCTTTGATGACCGTTTTTTCCGTTGCGCCATCTTGGGCAGAACAACCGGCA

AATACTGAAGAAATACAACCCGTCAAAACCTTCTCCCCGCCCAAACCGATTGCACCGACC

GCCGCACAAGGCTATTTCCCCGAAAACCAATTCGACCGCTCCGACCGCAGCGATTATTAC

TTTGTTACCGAAAACATAGACCAAGCCTTCCGTCCGCTGAAGGCAAACAGCAGTTTTTAC

GGCAAAAGCTTTTACAACTCCGTTACCGCGCAAGCACTCGGGGCGAAGGTATACGGCGTA

GCCAACCTCAATCGCACCAAGGCAAACGGCTACAAAGATGGCGGTGGGCGCGACACTGAT

TGGAAATACAGCCGCTTCAATCAGGCTTTGGTACTCGGTTTCGTGCCGTCTGAAAATCAA

GAATACCGCCTCACTTATCTGCACGACGACATCAACAACGACCGCCAGCCGCAGGTCGTC

AACGACGCATTGGACACCGAACGCCACATCTCCAAACTCAACGTGCGTTGGGGCAATGCC

GATTTGAGCAATACGGTCAGCGCGGAAGCAGGCGTCATCAAACTCAAACGCCATGCCGAC

AATTACTCCTTGCGACCAAACAACACGCCGCAGCAAGTGTTCGTAGAACTTGACCGCAAA

GTGTATGATTT

>FA_1090_pDFB12

ACCTGCCGACAAAACCTACCGCGTCGTCCTGCCCGTTTCCGCCGATGCCGAAAATCAGGC

GGCGGAGCTGTCTGCCAAAGGTTTCAACCCCATACCGTTTGACGGCGCATTGAGTTTGGG

TGTCGGCAACAGCCGGGAAAACGCCCAAGCCCTGCAAAACCGACTTGCCGATGCCGGATT

CGGCGGGGCGCATATTGTCGAACACTTTGCCGAAGCCGACAGGCAGGACGATTCTTTGAG

CGTGTCGCGTATGACGGTTTTGTTTACGGGCGTGAATGCCGCCGATGCGGACGAAATCCG

TAAAATCACGTCCCTATACGGCAAACTGAACCTCAAGTCTTGCAAATAAGTGCAGACCAA

AAACGCCAAACCCACCGCTAGGGTGGGTTTGGCGTTTCGGTTTTGAGTATCTGATTAAAA

AGGGAATGTATGTCTCGTATATGCCGACTCCAAGTGTGAAAGTGAATTGTGAGGGAATAC

AGTGTTTAATGGTTCGGAACGGACGGCTTCCGAGTGGTTTTGATTATACGCTAATAATAA

TAATTATCAATAAAAATTTAAAAATAAATGCGGATTTTTTGTAATGCGATGAAAAATAAA

CAAATTAACCTGTCCGCCGATGTTTGTGATTTGGGTCGGCAAACTTTCATTGCGCCGGAC

AAATCGGGGTCGGCAGGCATGGACGGGGGCTTCGTGCCTGTCGGAAAGAAGCCTGCCGTG

TTTGAACAACCGTCCGATTTGGTGCGGACAGGGTTGCCATTCGGGGGTGTTTGAGCGCGG

ATTCGGGGATGCAAAAGCGGTGAAACCGTTGCGGATGAAGGCTTACGGATGCCCCGAGGT

ATTTTTAAATGTATTCAAACGACAAAACTCCCTTCTATTGCCGTACTTAGGGCTTGCG

>FA_1090_pDFB15

CCAGTATCTCGCCGAATCCATCCGTATGCACCCCGATCAGGAAACTTTGAAACAGATGAT

GCTGGATGCAGGCTTCGACAGCGTGGATTATCACAATATGAGTGCGGGCATCGTCGCGCT

GCATAAGGGCGTGAAATTTTAAACGGACTGCCTGTGAAGCCAATGCCGTCTGAACACGTT

TCAGACGGCATTTTTGTATTTTTTGAGACAAAGGTTTTAAATCTTAAAAATTAATTCATA

TATTTATCAATAATTTATAAACTTTTTAAAAAATAGGAACAATTATCATTTGTAAAATTG

GGAGATGTCTGTATAATGCAGTCAATCCAGTAAACAACGCAGCAGACGAAAGGAGGGAAA

AATGCCGGAAAGTATTTTCAAACAGATTTCCTCCGATATTTTGAGGCTGCACCGTGATTC

TGTTTATTCCCTGCTAGCAACCTCGGGTTGCAACTGTCAGGTGCATGAAGCGGCATATGT

CAATATTGACGGCAAATATTATATTGCGCTTTCGTGCGAACCCGAGGTGGGGGAAGTTGA

AACAGGTATTTTGTTGATTGAAGATGAAAGCCGCAACCTTCGTTTGAGCTGGGTCGGTAG

TGCGAGGGAGCTCGACCGCAAGGATAATGCCTACAAACGCGCCCTGTCCGCGTTGTCCAG

AAAGCTGGGGCGGTGTAAGGACAAGCTGCATACGGCGGTTCAGCCGTTTTTGTTGGAGCT

GGTGCCGGAGAAAGGCAGATTTTCTGTCGGTGATGAAGAAGTTTGGATTTCTCGAAACGA

TTTAGTGAGGGCCTTATATCCCGTCGGGTACAGTATGCGGCAGGCAGTGTTGCAGATTTA

AAGTTTTGGTAGTGGTTTGTGTTCCTTTTGCGCCCCTTTCTCAAGGGGCGATTTTTTTTG

CACGCGTGTTGGCGGCAAAGGAAAAATGCCGTCTGAAACCGGTGTTCGGACGGCATCCGC

GTGCGGAATTACCTGTCCGGTAAAAGACGGATGCCTTGATTG

>FA_1090_pDFB16

CGCCAGCGAGATTTGGGTGGAAAGCCGCGCGCCTTTGGTGTTGATCGGGTCTTTGATGAC

CTGCACCAAAACCGACTGCCCTTCAAACAGCATATGTTCGATGCGCTGGGTTTCTTCGGG

GTTGCGGCGTTGTTCGAGGACATCGACGATGTGTAAAAACGCCGCGCGTTCCAAGCCGAT

GTCGATAAACGCGCTCTGCATCCCGGGCAGTACGCGGCGCACCACGCCCAGATAGATATT

GCCGACCAGGCTGTGCCCGCTGTTGCGCTCGATGTGCAGCTCGCAGATATTGTTTTCCTC

CAACACCGCCACGCGCGTTTCCTGCGGCGTGATGTTGACCAATATCGTTTCGGGCGGGCG

CGCGATGTCTTTGGGGATGGGGAGTCCTGACAACATGGTTTTTCCTGAAAATGTAATAAA

AATATTTTTGCATCCGTTCCGCCCCGCCGCGCAGAGCGGGTGCAAAAATATTTTTCACAA

TGCCTATCATACTTTAAAAAAGAAACTTTGACACACTCCGCACATGCCGTCTGAACGTGC

ATCTGTTCCGCACTTGCCAAACGGAGCGATTGCCCCTATATTGATTATCATTGCAAAACT

TTCGGAAACCCAATATGCAGACCGTTACCATGTACACAGGTCCGTTTTGCCCCTACTGCG

CGATGGCGAAAAGGCTGCTGCACGCGGCAGGTGTCGGACATATCGACGAAATCCGTGTCG

ATGCAAGCCCCGAAGCCTTTGCCGAAATGCAGCGGCTTTCGGGACAGCGCAGCGTGCCGC

AGATTTTCATCGGCGAAACGCACGTCGGAGGATTTACCGACCTCTACCGCCTTCAGCAGG

AAGGCGGGCTGGACGGACTGCTGAACCCTTAACCCACAACTAGGAAAACAAAATGAGCG

>FA_1090_pDFB17

CAGCTATGTTACCGGCCTAATGATGCCGTATTACGAATTGGCGGATGAAGAAGATATTGC

CAACCAAAAGCGGATGTTGCAGCTTTATGATCCCGATAGAGCTGATGGGCAGGTTGCAGA

ATATCAAGACCTTAACCGCCGTTTTAAGGAAAAAAACGGTTACGATTTTGATCCGCATGA

TACTCTCGTTGGTCAGGATACCCGGTTTTACGCCAAATCTGATGGAAATTTTGTTCAGTA

TGCGGACGGCAATAAAAATACCGATGTACTATATGTACTGCGCCGCAAACAGATCATTCC

AATGAATGCAGGCAAATTTGACCCCGGCAAAGTACAAATTACGCCGGAAATGTACCGCGA

AAGAGTAAACAATCCGCAGGGGAAAAGCGGCAGCTATCGCCGTTATATCCCAGGATCGCA

TATCTACATAACACCCGGCTCGGTACGCGAAGAAATGGAACGTATGGCCAATGAAAACCA

AATTCCGTCAAAAGAAGACAACAATCAGGCACTGGAATTCAATAGCGACCTAAACCGTAC

CGCTTGGATCCGGCATCAATTCGGCGAAGCCGACCGCTGGCGCATGCCGCAGGAGCAGCG

CGCGCATTCCTGGTCGCCCATGCTGGCGGTCAGCTACGATCTTGCCGACAACCACCGCCT

CTTCGCCCGCTATGCCCGCATGAGCCGCTTCCCCAGCCTTTACGAACTGACCGCCGCCAC

CGGTAGCGGAGGGCTGTACGGCAGCGAGACCGTGGCCGAATACAGCCTGAAGCCGGAAAA

AAGCACCAACTGGGAAGTCGGCTACAATTTCAATTTCGCCCCGCATTTCGCCAAACTGCG

CCAAGGCGACCTCCGCCTCACCTACTACAGTAATAAAATCAAAAACCAAATCGATACATC

CAATGAAGACGGCGGTATGATCCAATACGACAAGGCAGTCAGCAAGGGGGTAGAACTGCA

GAGCCGCCTCGACAGCGGCCGCTTCTTCGCCTCCTTCGGCGGCACGTACCGCTTGAAACA

TATGGTGTGCGACAAGGGTATCGCCTTCAAATTCGACTATTACCTGCAACGCGTACCCGA

ATGCCTTGAAGGTGGCTTCGGCCTCAGCCGCTTCTTCCAGTCCTTGCAGCCGAAATATTC

GCTTACCCTTGATGTCGGCACGCGCTTCTTCAACGAAAAACTGGAATTGGGCATGCGCGC

CATCCATCACAGCAAGGCGGAGCGGAGAAATTACGACAAGCTGATCGCCGACGGCGCGGG

GCAGGTGTATGCCCGCAACGGTAAACCCTACGGCTGGCATGCAGCCACCCTTTTGGATGC

CTATGCCCGCTACCGCATAGGGAAGCATATAGACTTGAACTTCAGCGTAACCAACCTTGC

CAACCGCTATTATCTAGACCCGATGTCCAGCACCCCGGTCCCCGGCCCGGGCAGGACGAT

TACCTTCGGCATCAAGGGCAGGTTTTAGGGTTTGACCGCATCATATTGCAGCCGGCGGAC

AGGAATCTGCCCGTCGGCTACAGCCTGTTGCCGTTACTATGATATTTGTATTTATTAGAT

GATAATGCAATGATATTAAAAATAAAATATTGACAAATGCAATCTACAACCAATATTTGA

TAATGAGTTTTAATTAGATTATTATTTGCATTCGTACTAATATGCACCCTGTCGGCAACA

GCCGATACATCTTGCCGCTGAAGACAAAACAACGGTATCCATCATATCTTTTCTTTAAAA

GGAAAAATCATGAAAGCATCACAATTAACCCTCGCCGTCCTTCTGGCCGCTGCTTTCGGC

TCTGCTTATGCAGTAGAAGTTAAAGGCGGGGATTCAAGTAAAGGGCAATTAATTCAAGCT

GCCGAATCCGATTTCCTACCCTTTGGTTCGGGTGCTGCCGACATCAAGGTCAGCACAGGC

AACGGCTTGTCCAAATCCATCAACCTCGAAGCCGGCCCCGCGCAGCGCATCCGCAACAAA

TACGGCAATGCGCCTATCAACGGCGGCAATCAGAACACCAATGTAAACGGTGCGGCCAAT

TCGAGATATCTGCAACCCGGCGACATCAACCCGATTGCGGGCTGGTTCTCGAAAACTAGA

CTCGCCCAAGTGTGGTATGAAAAACGTGCCAACAACACCGAAGTGTTCAGCGTACGCCA

>FA_1090_pDFB18

TTTTCAAGTTGTTTTGGGCAAAAATATGCGGCAAAGGCGGATCGGTGAGGATGTCGGTAC

TGCCGGCGGCATTGCGTTCGGCGCGCACCATAACCATACCGGCAGACAGCATAGTCGGAT

TTGGGGGCGTCATAAACGGCGAACCGCTGTCGGCTTCAAAATGGTCGGGACTGCCGACTT

CGCCCCACAGGCTGTGGTCGAGCATCAGGCGCCCCGTGATATTGCGGATGCCTTTGTCGC

GCAACTGGCGTTGGACGGCAAGCAGGTTTTCCTGATTGAAAACGGGGTCGCCGCTGCCCG

CCCAATACAGGTTTCCGTCAAGCGTGCCGTCGTTTACCGTACCGTTGCTTTTAAACTCGG

TCGCCCAGCGGTAATTGCTGCCGAAGGTTTTGAAGGCGGCAAACGCGGTAACGAGCTTCA

TCGTGGACGCGGGATTGACGGGTATGCCGGCACGGTGGTCAATGATGACTTTTCCGCTGT

CAAGCTCTTGGACATATACAGCGATTTCGTTTTGCGGAATGCGGCCGGTATCGAGCGCGT

GTGCGGCGAGGGAGGCGAGAAGCAGCAGCAGGGAGGCCGCTGTTTTGGGGAAATTCATAG

GTGAATCTGTTACATAAAAAAAGCAATTATAAGGCAAAGCCGGATAAGTGGGAACGCCGG

GGCGGCGGACCGGCTTGTTTGCAGGGGAAATCACATATATAATAATCGTTACCATTATGA

AATGATTGAAACGCACAAACTTAATATTCAGGAGGAATGATTGTGGCTAAGAAAATCAGT

ATTTTGGTGGGCAGCCTGCGCCTGCTTCGTTTGCGCGCAAAGTGGCATTGAATGCGGCGG

AGATGTTCCCCGAAGGCTGGCAGGCGGAAATCGTCGAAATCGGACATCTGCCGCTTTACA

ATTTCGATTATGACGACCCTGAGGTGGAAGATGTGCCGCTGCCCGAAAGCTACACGGCTT

TCCGCGAAACGATTAAGGCTTCGGACGGCATTTTGTTCGTTACGTCCGAAAACAACCGCA

CCATTCCCGCCTGTTTGAAAAATGCGGTGGACATCGGCTCGAAACCGAATGCCGACGTGG

CTTGGAAAAACAAACCGGCCGGCATCATCAGCCATTCCGTCGGCAAGATGGGCGGTTACA

GTTCCCAAAAAAACCTGCGCCTTGCCCTGTCGTATTTTGATATGCCCGTAACCGGACAGC

CGGAAGTATTTTTGGGCAATTCGCCGACGCTGTTTGATGAAAACGGTAAGTTGATTGACT

CGGCAAGGGATTTTGTTCAGTCATATATCAATCAGTTTGTCGGTTTGATTGAAAGAAATG

CCAAATAAACAAACCAAATCGAAAAGCCGCAAAACCGGTTCGGGTTTTGCGGCTTTTTTA

TATGGGCCGGGCAGCGTCAAGGTTTGCCGTTTGCCGGATAATGCGCGGCAAATGATCGGC

GTGTTTAAAATCCCGCGTGTACGGCACGCCCCGCGCTCCGCAATGATAGGGGGCAGTTTC

GTTTGAATCGGCGCACCGGGACGGATGCCCCCGCGCCGTCCCGATACCGGTTGTTCCGTA

TCGGTTGCCGCCGTGAAATGACGGACGGATGCAGAAATGTCGGCAGGCGTGAATG

>FA_1090_pDFB19

GGCTGCCTTCTTCTTTGATTTGGCCGGCAAGCTGGTCGCCTTTGGCACTGTTGAGTTTGA

CTTTGATGCCGGTAGCCCGGGTAAAGGCATCTGCAACGGCTTGTGCCGCTTCTTTGTGTT

GGCCGTTGTACACGGTAATGTCTGCCAGCGCGGGGGTGGCGGCGGTCAGGGCTGCGGCAA

GCAGTGCGTATCGGATAGATGTTTTCATATCGATTTTCTCCTAAATGAATGAGGGTGTAT

ACCTTGTTAAGACATAACGGGGTGTAGTGTATTCCTTCTTTTTTATAAATGCAAATAATT

ATTTTTTAAATTTGTTATTATCCGATCCGGTTATTGTTTGTTCTGACTTGTATTTTTTCC

GTGCATCGCGCCCGTAAGGCGGAAGCGGCGGGCAATGCGTGGCGGAATGTGGGTAAAGGC

GGCATTTTGATTTGTCGGAATGCTGGAGAACCTCTCCCTTTAAAACGCCGTCTGAACAAG

GTTGCCGGAATAGTATTGCCATCCCGGCAGATACAGTTTGTCGGGATCTTGCCAATATTG

TTCATCCAGACTGTTCGGCAGCGAGGCGGTTTTGTTGTCGAGATGTTTTGTTAATCCACT

ATATATCCCACATTTCTTTTAGGTTTTTACCTTCCGATTGGA

>FA_1090_pDFB1

GGGTCGGAATTTTGCGGCGAACACGCGCCCAAGGCGAGGACGGTGCAGAGGGTCAATGCG

GTCAAACGCAACATAGGGTGTCTCCAAAATGGGGATATTGGGGCAAAGCCGCCGGTCGGA

CAAACCGGAACGGCTTTAGAAAGGATAAATGATAATCTATATCAAATTATCAGGACAGAT

GCCGTCTGAAAGGCTTTCAGACGGCATTTTTTCGGGATGTGCGTTTTAGAACTTGTAGTT

CACGCCCAGGCGTACATCACGTCCCACGCCCGGCAGGGTATTGGTCCAGCGTTGGCTGTG

CGGATAGTAGAACTTGTCGAACACGTTGTTAACCGAAAGATTAACATTGAGCGTGTCTTT

GCCCAGCGGTTTCCAGTTGGCGAAGACATCGTTCACACCGAAACCTTGGCGTACAACGTT

TTCCAATTTGCCGTCGCGGTCTTTTTGCCCTGCCGCCAATATCGAACCCGTAGCTTTTTG

AACATAGCGTCCGCGCCAGCCGATTTCCAGATTCGGGTTTTTGAAGCGGTAGGCAAGGGA

GGCCGTCCAAGTGCGGCCGGTTTGTGCGCCAAACTCGGGGTTCGCGCTCAACAGTTTTTT

AGGATGGGTATCGTAAAAGCGCGGTTTGCTGCGGCTTACGCCGACTTTGGCGGTCAGGCC

GCCGGTGCGGTAGGACGCGCCCAATTCGTAACCGTGGTTTTTGATGTAGCCGGCGTTGAC

GGCTTCGCGGACGGCGACAGAGTCGTGGCGGTTTTGCGGATTGGCAAGCGCGTCTTTGAT

GGTCTGCCGGAAGTAGCTGCCGTTTGCGGCAAACGTGCCGTCGTTGTAGTTGAAGCCGAT

TTCGGTATTGCGCGCGCGTTCGGCTTTGGTGCCGTCGGCAATCGAGATGATGCCGCGTTT

GCCGTGGGTTTGCAGCGCGTCATACAGGCGCGGGCTGCGGCTGGCGTAGTTGTGGCTCGC

GCTGAAGCTCCAGTGTTCGCGCGGCTGCCAAATCACGCCGAAACTCGGGTTGAGGCTGCT

GCTTGAAACGGTTTTGCCGTCGTGGGTTTTCACCTTGAAGCGGTCGTAACGCAGCCCGCC

GGTCAGGGTAAAGCCGTCAATCTCGTGAATGGCTTCGATATACGCGCCGGTATCGGTTTT

GGTCGGGTTGGTCAGACGGTAGGCTTTGGCAATTTTTTCATTTTCACGGTTCTTCTTTTT

ATCTTCCTCAGTTGCATCTTTTTTATCTTCAATTTTAAATTGTGAATTCAAAAACGCTTG

CGGTTTGATTTCCTGATGGCGGTAGTTGATGCCGTATTTCAACAGGGTTTGTTCGGCA

>FA_1090_pDFB20

GCAGAAATGGCGGCGGCGGTGCTTCTGCCTACGCCGCAGAGCGTTTCCAAGTCTTTGCGC

TCCGATGGAAACGTACCGCCGAATTGTCCGACGATTTGTTGCGCGGCTTTGTGCAGATTG

CGCGCGCGGCCGTAATAGCCCAAGCCCGCCCACAACGACAACACTTCGTCTTGCGGCGCG

GCGGCAAGCGTCTGAACGGTCGGGAATTTTTCCAAGAAACGCGGATAGTAGTCCAACACG

GCGGCGACCTGCGTTTGCTGGAGCATGATTTCGGAAAGCCAGACGCAATAAGGGTTTTTG

ACCTGCCAAGGGAGGTGGTGGCGACCGTGTTGTTTTTGCCAGCGGATGAGCCGTTCGGAG

AAGGGGATGGGTGTGTTCATTAATATCAATCGGTGGTTTTATTTATATTTAAAACAGTAT

GTTATTGTATAAAATTGTGAAAATAATTCTTATTGACTTATTTTTTGTAGGGGCATATAA

CTCATATAAAGAAACTTTATTGTGGTATTGAAATTATTTATCAACAAGCAAGGAGTATCA

GCATGAAAGCAATGGTTTATCACGGCGCAAACGACATCCGTTTTGAAGAAAAACCCCGCC

CGCAGATTATCGATCCGACCGATGCGGTGGTGAAAATCGTCAAAACCACGATTTGCGGTA

CCGACTTGGGTATTTGGAAAGGCAAAAACCCCGAAGTCGCCGACGGTCGTATCCTCGGTC

ATGAAGGCATCGGTATTGTAGAAGAAGTCGGCGAGGCTGTAAAAAACATCAAAGTCGGCG

ATAAAGTCATTATTTCATGCGTCAGCAAATGCTGCACTTGCGACAACTGCAAAATCCAAC

TTTATTCGCACTGCCGCAACGGCGGCTGGATTTTGGGCTATATGATTGACGGCACGCAGG

CCGAATACGTCCGCACGCCTTATGCCGACAACAGCCTCGTGCCGCCGCACGACAACGTCA

ACGAAGAAATCGCCCTGCTGTTGAGCGACGCTTTACCGACCGCCCACGAAATCGGCGTGC

AATACGGCGATGTGAAACCCGGCGATACCGTATTCATCGCAGGTGCCGGCCCTGTCGGTA

TGTCCGCCTTGTTGACCGCCCAACTGTATAGCCCTGCCGCCATTATCGTGTGCGATATGG

ACGAAAACCGTTTGAAACCGGCGAAAGAGTTGGGCGCGACCCATACCATCAGCCCCGCTT

CCGGCGACGTCTCCAAACAAGTTTTCGCCATCGTCGGCGAAGACGGCGTAGATTGTGCCA

TCGAAGCGGTCGGTATCCCTGCAACTTGGAATATGTGCCAAGACATCGTGAAACCCGGCG

GTCATATCGCCGTCGTCGGCGTACACGGTCAATCCGTTGATTTCAAACTGGAAAAACTGT

GGATTAAAAATCTTGCC

>FA_1090_pDFB21

AAAGGCTCGTGCGGCCAGTTGTTGGTGTAGGTAAAGACCTCGCCGGGACGGTTGGTCGAA

GCAGACCAAGAAGTCCAGAAGAAGAAGTCGAACAGTTTTTCACGCGCTTCTTGGCTTGGC

AATGTGTTGTTTTTCATTGCAAAGTGTTCGCGGGTGGTTTGCAACTTGGGATCATCGCCG

TAAACGCCGTGATAGTAAGGCAGGATGCTTTCGATGGCTTTCACGCGCGTATCGCTGATG

ACGACGCTGCCGTCTTCTTTAATACGGCTTTGATTGCGGTATTCATCGGCCAAGCGGGTT

TTCAGAACGGCTTGTTCTTCAGGGGAAACTTCATCGAATTTTTTGCCGTAAGTCTGTTGC

GCGGTCAAATCCAACCAGGCGGACAACTCACGATGCAGCCAGTCGGCCGTCCAGTCCGGA

GCCTGATATGCGCCGTGACCCAGAATCGAACCGACTTCCATGCCGCCGGTACTCTGCCAC

GCAGACTGACCTGCCAAAATATCGTCTTTCGTCATCAGCACTTTGCCGGATGCGGAAACG

ACCTGTTCGGGGTAAGGCGGGGCTTTCTTATAAACCTCGCTGCCCATATAGCCAAGAATG

GTAAAGCATACCGCCAGAACGGCAAACAGCAAGTACCACAGCTTCTTGTACTGTCCCATT

TTGAGAGCTCCTTTTAATAAATCAGTTGTTTAAAATTCACAAAATATGAATGTTAAAGAT

TGTAGCATGGTTTACCGCGCAAATAAACATTTGTTCAAACAAACTCACATATAGAACAAA

TACATATATGATAATAACTATCATTATTCTTTACTCGGAAATTGCCCTGCCTTTGCCTGC

TCTGCCGGAGCCCCTAGCAAATCAGCCTATTCATTGTAATTTTTAGTAGTTATAAAGT

>FA_1090_pDFB22

CGACTGCCTGTTGCATATCCGGAGGCGATTCGTTCAGGTCGGCGGCAATTTCGGGCAGTG

CGGTATTTAAAATGGTCGCATCCAACATCTGCATAAAAATGGCAATTGCCAGCAGAAGCG

GCAGCCAAGGGGATGGTGCGCGGGCGGACAGGGTGTTTTTTTCCATAGGGTGATTGTACC

CCATCCTTGTGCCGTTATTGTTTTCAGATGCTGTCTGAATGCCGGCAGAGTCGGCATCCT

GAATGTTCACAAGCAAACGAATCGGCATTGCATTGTAATGATAATTATTATCGAAAATCA

TCAGAGTAAGGTACAGTAAGCGTTATGGGGCAGTTTGTAAGAAAAACCGGATTATTTTTT

AAAATTAGACTTGACCCGCAACGGTCAATTACTTAAAGTAAACGCTTACCTTTCTACAGA

AAAAACGGGTTTCCCGTTATCAAAAAATATGAGCGCAACCATTCCCCCAAAAATCATCCG

ATACGACAGCAATCCGACAGATGTCTATTTTTTCGGCACTTGCGTCCTTGATCTTTTTAT

GCCCGAAGCAGGCATGGATGCCATTGCCCTAATCGAGCAGCAGGGCATACGCGTCCATTT

CCCGATGGCGCAAAGCTGCTGCGGTCAGCCTGCCTATTCATCCGGCCATCCGACCGAAGC

CTTCGATGTCGCCAAAGTGCAACTCGACCTTTTCCCCGAAAACTGGCCGATTGTCGTACC

GTCCGGCTCATGCGGCGGCATGATGAAACATCACTGGCCTACCCTGTTTAAAAATACAGA

ATACGAATCCAAAGCGGTTGATTGTGCCAACCGTATCATTGAGTTTACTCATTTCTTGCT

TGCCATCGGCTACAAGCCTGAAGACAAAGGCGAGCCAGTCAAAGTCGCCGTTCACACTTC

CTGTGCCGCCCGCCGAGAAATGAATGTCCATCTTTCAGGCTGGCAGTTGATTGACGGTAT

GGAAAACGTCGAACGCATCGTCCACGACCACGAAAGCGAGTGTTGCGGCTTCGGCGGCAC

ATTCTCCGTCAAACAAGCCGATATTTCCGGCGCGATGGTAACCGACAAAGTTGCCGCGCT

GAAAGAAACCGGCGCAACCGAAATCATCAGCGCGGACTGCGGCTGTATGATGAACATCGG

AGGCAAAATCGCCAAGGACGAGCCGGATATGCCGCGTCCGAAGCATATCGCATCCTTCTT

GTTGGAACGCACCGGAGGCAAAGTATGAGCGCGCGCGAAAATATTTTGGCAAAACTGAAA

AAAGCCGGCGCATTGCCGATGGAAGAGCCTGCGGTTTTTGATTATTACCGCGAAAAGGGC

GTTTCTTGGGACAGCGAAGCCGAGCGTCTGAAACATTGGGCTGCCGCCATGCGCGCGGTC

AAAACCGAAATCTATTGGGTGACGAAAAGCAATTGGATGCAGGTTTTCCGCGAAGCGGCA

GAAGGCAAGGGTTTAAAAAACATCCTGCTGCCCTTGGCAACCGAACACGGACAAATTGCC

CGTGCCGCATTGGCGGGCAGCAATATCGACCCGATTGCCTTCGAGCGCGAAATCGATACT

TGGAAAACCGAGTTTTTCACGAACATCGATGCAGGCTTCAGCGGCGCGCAATGCGGCATC

GCCCGCACCGGCACGCTGATGCTGTTTTCCAGCCCTGAAGAACCGCGTACTTTGAGCCTC

GTTCCGCCCGTGCATTTCTGCCTGTTCGATACGTCCAAGATGTACAACGAGTTTCACAAC

GCCGTCGAAGGCGAAAAACTGGTGGAAAACGGTATGCCGACCAATGTATTCCTGATTTCC

GGCCCGTCCAAAACCGCAGACATCCAGTTGACGCTCGCTTACGGTGCGCACGGTCCGCGT

GATTTGGTCATCCTCGCCATTTTGCCCGACCACATTTCCCCTGCCGATTTGGAGGAAAAC

GCATGACTACGCAAACCATCAAGTTCCACATGAAGCCGGAAAC

>FA_1090_pDFB23

CCTTGTTGACTGGAATCTTGCAATGGTGCCTCCCCATTTCCAGATAAGGCATCAATAATA

TTTTTTTGTTTGGAAGGGTTTTTGATATTACCATTACCCGTTTGTTCCCAATCACCTTCA

GAGAGTCTTATCTCATTTTCTTTAGGATTTGACGGCGGATACCAATTGCGCCGCTTGAAG

CGCATCGCGAAACCGTATCCGCCTTGGTCTTTTCGGGCTTCCGGTTTTTTGGAAGGAACA

TCTTGATACTTTGGCGCGGGACGGGGGGCTTCGGTATCGACAGAATCAAGATCGAAACTG

CCGCCTCCGCCCAGACAAGCGCTCAACAAAAACACAGGCAACACCATAGCAGCCTGATTC

ACCAATGGATTGTTCATAACAAACCCGATTCAATTAAAGAATGATAAGGATTATTATTTT

ATTTATTTTTAAAAAATTTGCAAATGCTTTTTTATATTTTTAGTAAATAAACCAAAATAC

CGTCATTCCCGCGCTGCTTTTAAAACCGCCTGCAACCGCCAAACCTGCTGCAATCCGCTC

CCTCCCCTGCGCGGCGGAGGGCTGGGGAGAGGGCACTTTCCAAGTTGCGGCAATCTTTCC

CAAACTCCCTTAGTCTTCTAAAACACAAGCCTTGCGGCTTGTTGCCCTCTCTCTAGCTTT

CTCCCGCAGGGAGAGAGGACGGTGCGGCTGTTGGGGTTAAGGTTTCTGTAAACTAATCAG

CTTGTGCAGGCTGTTTTTTATTTTTCATACGACCTGAAATCAACGGTAAATCCAAGTTGC

TTTTCAAACCGCCTGCAACCGCAAAATCTGCCGCAATCCGCTCCCTCCCCCGTGGGGGAG

GGCCGGGGAGAGGGCATTCTCCGAGTTGCGGCAATCTTTCCCAAACTCCCTTAGTCTTCT

AAAATACAAACCTTGCGGCTTGTTGCCCTCTCTCTAACTCTCTCCCGCAGGGAGAGAGGA

CGGGGTGGCTGTTGGGGTTAAGGTTTATGCAAACTAATACGGCTTGCTGCCCTCTCTCTA

ACTCTCTCCCGCAGGGAGAGAGGACGGGGTGGCTGTTGGGGTTAAGGTTTATGCAAACTA

ATACGGCTTGTGCAGGCTGTTTTTTATTTTTCATACGACCTGAAATCAACGGTAAATCCA

AGTTGCTTTTCAAACCGCCTGCAACCGCAAAATCTGCCGCAATCCGCTCCCTCCCCCGTG

GGGGAGGGCCGGGGAGAGGGCATTCTCCGAGTTGCGGCAATCTTTCCCAAACTCCCTTAG

TCTTCTAAAATACAAACCTTGCGGCTTGTTGCCCTCTCTCTAACTCTCTCCCGCAGGGAG

AGAGGACGGGGTGGCTGTTGGGGTT

>FA_1090_pDFB24

CCTTGCCCGTTTCAAAATGGTGGCGTTGCAGAATGCCCGCCTGCTCGAACTGCGTCAGCA

CGCGGTAAATCGTCGCCACACCGATTTCCACGCCCTCTTCCAGCAGAATGCGGTACACAT

CTTCCGCACTCAAATGCTCTTCCGCGTGCTTCTCGAACAAATCCAAAATCTTCAAACGCG

GGCCGGTAACCTTCAGACCGCTGTCTTTCAGTTGCGCAATGTTGCTGAATTTTTCCATAA

TATTCAATATCCCTGTAAAATAATAGACGCTATAATACGCAATTTCAGCCTGCTTGCCCA

CTATCACACCATAGCAGTTCGCAATAGCAAAACCGCAACGGCGGACGGGCAGTCGGACAC

AGACAGGGTAAAATACCGCTTAAGCGTATGATTATCGTTCGCTTTTATAAAATATTCAAG

CAGTGCTACACTAGACATCCCGATTTGCACAGAAAGGTTCTCCCGTGAACAAAACCCTCA

TCCTCGCCCTTTCCGCCCTGTTCAGCCTGACCGCGTGCAGCGTCGAACGCGTCTCGCTGT

TTCCCTCCTACAAACTCAAAATCATCCAAGGCAACGAACTCGAACCGCGCGCCGTTGCCG

CCCTGCGCCCCGGCATGACCAAAGACCAAGTCCTGCTCCTGCTCGGCAGCCCCATACTGC

GCGACGCTTTCCATACCGACCGCTGGGACTATACCTTCAACACCTCCCGCAACGGCATCA

TCAAAGAACGCAGCAACCTGACCGTCTA

>FA_1090_pDFB25

GCGGTCGGGAATTTTGAAATCGGGGGAGGAGTAGCTGACGGTTATGGGGTCGGCGTAGGA

TGAAAGGGCGTATTGCGCGCCGCCGGGTGTTTTGAAGATGAATGATGTTTCTTTTGATTT

ATAGCCGAAATAGTCTTTTTCTACTTCCCGCTGCCGAATGGAAATGCCGGATGTGTTTTG

CGGGGCGGCATCGAGCGAGCGTCTGCTGCGGCTCGGCGCGTCGGATGCGCTTCCGGCGCC

GGCGGTGTTGATGTTTTCGATATTGCCGTTATCGCTTGATAACGTTACCTCGCCGGCGGG

CAGCGGCGTGGCGGTGGGGATGGACACGGGGACGTGCGGTTCGGCTACCCCCCCCCGGCA

CAGGCGGCAAGGGCGGCGGCAAGCGGCAGTAAGGGCAGGGCTTTGTATTTCATCGCATAC

TCAATCTTGATGATGTATTAATAATAGTTTTGATTATCAATTTAAATGTTATATGATGTC

AACTGTTTTATGGGGCGGCGCGGAAATCGGACGTGCCTGCGTTTGTCTGCCGCGTTTCTA

CCTTTGTTTTTTATAGCGGATTAACAAAAACCGGTACGGCGTTGCCTCGCCTTGCCGTAC

TATCTGTACTGTCTGCGGCTTCGTCGCCTTGTCCTGATTTTTGTTAATCCACTATATGAT

GCGCCGCGTCCGCCGCGCGTGGGGCTTGCGGCGTTTCTGCCGCCGCCGTCCGGCGGTGTT

TTTTAAGGAAGGCAAATTGAAGGATAGTGTGTATCGGGTATGCCGGCTGTGTGCCGCGCT

GTGTGTTTGGGGTGCGGTCGGCGCGTATGCCGCCGGCTTGCCCGACGTTCGGGACGATGC

GGCGGCGTTGCGCGCCCAGCGTGCGGCTGCGGAGGGTTGGGCGGGAATGCCGCCGGAAGG

GGATTCTGCCGCAAACGGCGGTTCGCGCGTAATCGACGGGGATTTCCTGCTGTCGCGCCC

GCAGTTGTTGGAACACGTTTTGCGCGACGCGCTCAACGGCAATCAGGCGGATTTGATCGC

TTCGTTGGCGGATTTGTATGCCAAGCTGCCGGATTATGATGCGGTGTTGTACGGCAGGGC

GCGGGCTTTGCTGGCGAAATTGGCGGGAAGGCCGGCGGAGGCGGTGGCGCGCTATCGGGC

GTTGCACAGGGATAATGCGGCAGACGAGCGGGTTTTGCTGGATTTGGCGGCGGCGGAGTT

TGACGATTTCCGGCTGAAGTCGGCAGGGCTGCATTTTGCGGAGGCGGCAAAATTGGATTT

GCCGGCGCCGGTTTTGGAAAATGTGGGGCGTTTCCGGAAAAAAACGGAGGGGCTGACGGG

CTGGCATTTTTCGGGCGGCATCAGCCCTTCTGTGAACAAAAACGCCAACAATGCCGCGCC

GCAAT

>FA_1090_pDFB27

CATCCTTGCGTTTTCAAAGGATTCAAAACCGCATTTGTAAGGCGCGTCTTTTTCGGCATA

GTGGCGTTTCGGGCCCAAAATCGTGCCGAGCAGGATAAACAGTACGCCGGCCGCGAGGCC

GACGAGGATAAAGACAAAGACGGGAAAATAAGCGGACAACATGGTTACACCCAAATCCGT

TAACAAAATTTCTACAATAATTTCGTATTTTAGCGAATTTCAAAAACCATTAAAAGGTAA

ATATCGGCAAAACGCCCAAAAAAACCCAATAAATACAATCATGTTATGATAACGATTCTT

ATTTGATTTTATAAGGTCATATAAATTTTTACACCGTAATTCGTGGTATAGCGGATTAAC

AAAAACCGGTACGGCGTTGCCCCGCCTTAGCTCAAAGGGAACGATTCCCTAAGGTGCCCA

AGCACCAAGCGAATCGGTTCCGTACTATCCGTACTGTCTGCGGCCCGCCGCCTTGTCCTG

ATTTTTGTTAATCCGCTATATATAGGAATAAGAATTCGGGACAAAATGTTTAAACCATTT

TGTCCCGACTGCTGTGATGCGGTTTTTTTGAACTAGCAGGACGTTACCTAAAACCTGCGT

TGCCTAAAACCTGCGTTGCCTAAAACCTGCGTTGCCTGAAACCCTTTAAGACGGTTAAAT

CCCTCTGCCGTATTTGTATTCTACAATCATATTATCGTCAGTAATGACTTCCGCCCCAGC

CGAAGGTTCCGTCATCCGAATCAGCATACGAGAGACAACCTTTTGTGCTGCAGCATCCAC

GGTGCTGCTGTCAAATACGTGCCTGCCGCTTTCCGGCCAAATCAACCGGGAAAGGCGTTG

CTTGAGCAGTTCTTTATTAGGGAAAACTACCGGGGTTGCCGAGCCGACTACCATATGCCC

GTAGCGGTATGCATAGGGAATACTGTGTACGGCGGTAGCAAAAGCATGCGGGCTGTGCGT

GGTATTAAACATTACAATACCATCCGGGGTAAGGTGGCTTTGCACCTGTTTTAAAAATTC

CGCACTCAACAGGTTAGTGGAATAGGCACGCCAGTACCAAGTCGAATTCATCAAAATCAG

GTCGAATTTTTCATCAGGATGGCGACGCAGCCATTTCCTACCGTCATCCAATACAATTTC

AACACGTTTGTCCTGCAAAAGCGGTGCGATTTGCGGCTCGTCCGCGATAAGGCTACGGTA

TGCCGGATTGATTTCC

>FA_1090_pDFB2

TTACATCTTCCTGCCTTGTCAATCAAATAAAGACAGAAGACTATACAAAAACTGACCGAC

ATACTGAAAATACCTATCCCCTTCCATGCAATCTCCGCACCATTGACCCAATAGATCAGA

CTGAGAAACAACAAAGCCACAGTATAAATCAACGTAAAAAATATAGCGTAAAGCGCAATA

AAGGGAACTTTTATCTCACGGTTGTTTTTTATAATATATTCAGCAACTTGATTTCCGAAT

ATACCTGATAAAAAATATAGTATTAGATCATCCATTTCGTTTATCTTCTATGTTTTCCCA

TTGCGGCGCTAGAAGACTGATTTAATGCATGCACCATGCAATATAGTGGATTAACAAAAA

CCGGTACGGCGTTGCCTCGCCTTAGCTCAAAGAGAACGATTCCCTAAGGTGCTGAAGCAC

CAAGTGAATCGGTTCCGTACTATTTGTACTGTCTGCGGCTTCGTTGCCTTGTCCTGATTT

TTGTTAATCCACTATAGTTTAAACAACTTTATTTTTGATTTTATCCCAAGTATAAACCTG

ACAATCGTATTTAAAAGCCGGATCTACGCCTAGCTTGTCCACTGGTATGTATGATTTAGA

CTAAAAAGAAATCAATCATTTGCTCAATTTTAAGCGTCTCCCCGACAGGTTCCCCTGTCT

CAGGATCAAACTCAAGAAAACCTGATAAAAAATCATTAATAATCAATAATTTTTCATCAC

TGGAAACAGTATTGTTTATAATTTTTTCCCTTAGTACGGATAAAGCAAAATTTTCTAAAG

AACTATATTGCCCCCCCATCAATTTTTTTAGAAATTAAAGCAACTATTTCATTTTTAATA

TTCATAATTTATCCTTAAGTTATAAAAGGGGCAAAGCCCCCTTTTTTTGATTAACGAGGC

TTTGTTGCGCGATTTTTCGTAAACCGTGCTTTCAAGAAAGCTGATTAGGGCGGGGTTTTT

GCCGTCTTTAAGGAAGGTGTGGCTGATGCCGGCGGATATGTCGGACATCCGTTTGTTGCG

GGTTTTGCCGTTGCCGTCGAGTTTGCGTTCTTCGTGCCACAGATAGCTGCCGCTGCCGTA

AATGTCGGTATTGCCGGTCAGTCCGTAGCGCAAACCGAGCGTGCCGGCGAGCATATCGGT

ATTGCTGCCGTTTTCTTGAATTTCGGTCGGAATGGGGATAAACGAGGTTGCGCCGGTTTG

AATGTAAACCGGTGCGGCAAGTGCGGCGCGGCTGTTTTCGCTATTCAGATAGGTAAGGGA

AGTTTCCAGTTTCCATTTTCCCTTGTCGGTCATTATGTCTTCAATCGTCAAGGGCAGGTC

GGCATAAGCGGATAAAGGCAGGATGGCGGGCAAGGCGGGCAAAAAGATACGTTTCATATT

TCCTCTTATTGATATTAATTCTTATTTAATAATAGTAATGAATATCCGATAATTCTGTTT

AAACAAATTTTTAAATCAAAAAAAAACGATATATTTGTTTTTATCAATTATGTTTTGATA

TGCCGTCTGAAAAGTTTGTGAAAAACGGTTACAATCCGCCGCATGAAAAAACGCAATAAT

CCTCTTCCGCTGTTGAACGGTGTCAAACCCAGTTATTTGGTGCTGCCGCATGAAAAGCAG

TTTTACGGGCTGCCGCTGCTGCATTTTCTGTGCATCCGCTTTCCTTTTTTGGGAGCGGAC

GATTGGCGGAGGCGGTTGAACAGCGGTTTTGTGGTCGGTTCGGATGGTGCGGCGTTGGAC

GAACATTCTTTGTTCGAGCCCGGTAAGACGATGTTTTATTACCGTGAAACCGGTTGTGAG

AGCGAGCCGCGTATTCCGTTTGAAGAAAAGATTTTGTTTGTCGATGAAC

>FA_1090_pDFB4

GGGGATATTCGGGTAAAATTAGGAGGTATTTGGGGCGAAAACAGCCGAAAACCTGTGTTT

GGGTTTCGGATGTCGAAGGAAGGGCTTTTTTGCAAAGGCCTCATGCCGTCTGAACAGGCT

TCAGACGGCATTTTTGCCGCGTGCCGGATGCGGAAACCAATCAGGCGTAATGTCGTGCAA

GAAAACCGGGCAGTTCGGACAAACCGTCCAATACGGCGAGATGCGGTGCGCCAAGGAGCT

GTTCGCGCGAATGTGCGCCGGTGGCAACGCCGACTGCCGCCGCGCCTGCGTTTGCCGCCA

TATGCAGGTCGTGCGCCGTATCGCCGACAACCAATGCCTCTTTCGGGTCGAGTCCCAGTT

CGCCGCAGAGTCCGAATACCATTTCGGGCGAGGGTTTGGAGGGGTATTCCCCCGCGCAGG

CGGTGGCGAGCCAATAGCCGCCGGTGGCGGTTTGGCTGATGGCGTTGTCCAAACCCGCCC

GCCCTTTGCCCGTGGCGACGGCAAGCCGGAAGCCTTGCGCTTTGAGCTTGTCCAGACAGG

GCGGGGCATCGGGAAAGAGTGTCATATTGCGGTTGTTGGGATTGAGGTAATGCGCGGAAT

AAGTGCGCGCGATGTCGGCAACGGCAGCTTCAGAAGGCATTTCGAGCAGGGCGCGGATGA

TTTCGGGCAGGCTGTATCCGATCAGGCTGCGGACGCGCTCCGCTTCGGGCGGCGGAAAAC

CGCATTCGGCGAAGCTGCGGCGCATGGTGTCGATGATGGGCTGGGTCGTATCGGCAAGCG

TGCCGTCCCAGTCGAAGATGATGAGTTTGGGCGGGGTCATGGCAGGTTGGTTGCAGTAAA

AAAGCAGATTTTATGCGGAAAACGCAGACGTGTCGCATTTTCGACAAAATTTGTCGGCTG

AGCGATATGTTTTTCCGAACAAGCCGCGTTGTGCTTTATTAAAATAGAACCATTATCATT

TATACAGATGGGACAGTTTATGTCAGTTTTCCGCATCAATATGACCGCCGCCACGGTTTT

GGCAGCACTCTCGTCTCCGGTTTTTGCCGCACAAACGGCGGATTTGGAAACCGTCCATAT

CAAAGGGCAGCGTTCGTACAACGCGATTGCCACCGAGAAAAACGGCGATTACAGCTCGTT

AAAGGCAGCTATATGGATGACCGCCTCAATACCCGCGTCTCCCCCTGCCGCCTGAAAGAC

AAAAACGCCGCCGAACCCGAACAACCGCAACACCCGTTACGCCGCATTGGGCAAACGCGT

GATGGAAGGCGTTGAGACCGAAATCAGCGGTGCGATTACACCGAAATGGCAAATCCATGC

AGGTTACAGCTATCTGCACAGCCAAATCAAAACCGCCGCCAATCCACGCGACGACGGCAT

CTTCCTGCTGGTGCCCAAACACAGCGCAAACCTGTGGACGACTTACCAAGTTACGCCCGG

GCTGACCGTCGGCGGCGGCGTGAACGCGATGAGCGGCATTACTTCATCTGCAGGGATGCA

TGCAGGCGGTTATGCCACGTTCGATGCGATGGCGGCATACCGCTTCACGCCCAAGCTGAA

GCTGCAAATCAATGCCGACAACATCTTCAACCGCCATTACTACGCCCGCGTCGGCGGCAC

GAACACCTTTAACATTCCCGG

>FA_1090_pDFB5

TGCAGGGTTTGGTAATTCACGCCGCCACCCAATGCGCCGCTACCGGTATTGAAAGAGTCA

GCGCCCTTCGCGATTTCGATGTTGCGCACGAGTTCGGGGTCGATAGACAGGCGCGAGCTG

TTGAAGTTGCCATAACGTGCATACAGTGAGTTTTCTTCCGAATCAGGCAGGCTCACGCCG

TCAATGCTGACACCGACACGGTTGCCTTCCACGCCGCGCACAGCAAAGCCTTTTTGATGG

CGGCCGCTATCGCTCAAGCCGACGTCGGTGGAGTAACGCACCAAGTCTTTGTTGTCGCGT

ATCATTTCCTGTTGAATGCGGTCGAGGTTGACACGTTCCACGGTTGCAGGCGCATTAAGC

TGGTCTTTAACGCGCACTTCTTTTATCTCTGCTTTAACGGGTGTGGTTTCGGTTGCAGCT

TCATCCGCTGCCAAGACCGGATTGCCGAAAATACTGCCGACCAGCGCGGCAATAGGAAGC

ATGTGTAATGGTTTCATATTATCAACTCAAGATGTAATGGATTGTTCATCCATCAGTTAA

TTCATAATAAATTTTCTGATAATCATTAATATTTAATAAGACAGCAACCCGTACAAATAA

ATCTGTGTCGTGTAATTAAAGGTCTCTGCAAACATCTATGCCGAGACCTTGTTTGTTTCA

TTATTTTTAAGGGTCTGTACTAGATTGGCAGATATGTTACCCTCGAAATATGAAGATAAC

ACACTGCAAATTAAAGAAAGAAGTACAGAAAGAACCGCTCCGTT

>FA_1090_pDFB6

TTCAAGTCTTGCGTTACGGCATCGTATCGCGCCATGTATTCCAGCTCGGAAATGGCTTTG

TTCAACTCGGGGTCTTTATAGATATGATCAACCGCTTTGTGGAACACGGATTGCAGTTTC

AAAAATTTGATGTAGTTTTCTTTGCTGACAAACGGTTGGACAGACATAACGAGGTTATCC

ACGCTGTCGTGAACCGCCGTGGTATCCGCCTTCAGGCGTTTGGCAAACGTCAGGGCTTGG

TTTTCGGTTTCGCTCATAAGTTCTCCTTTGTCGGTAAGGGATAAGGATGCGGTGCGGGCA

AAATCCGGACTTGCCCGCATACAAAAATAATAAAGAATCATAAACGAAATTTATTATCAC

ATATTTTACGAAAAAATATCATTTGCGTTATATTTTTAAGCGGGTATTTTATGATTATTT

ACAAAATCGGGGTTTTATCAAATAGGCTTGTCGGCCGGAGGGGCAGCCGCTCAAAAAATA

TTTTTGCCGGACACCAAGGGTTTGTTCATACTGCCGAACCTGCCGGTTTTGCATCCTGAT

TGGGTGTATCGCTTTTTTTCCTTTATAATGCCGCCATTTATATTTGCCACTTTCCCGATG

AAGCCGTTTGCCGAAAATATCCCCCACAGCCTTCGCGGCAACTGCCGCGACGAAGCCCTG

CCGCCGCATACGGTAGATTGCCCGGAATGCGGCTGCCGTACAGACGTGCCCCAGTTGGAC

AAGGGAGAGGCGGCGTTCTGTCCCCGTTGCGGACACAAACTCTTCAGGGTGGGCAGCCAC

CCTTTTTCCGGTCCGCCCGCCTATGCGGCGGCTTCGCTGATTTTGATGGCGTTTGCTTAC

AGTATGACGTATATCGAGGTCGGGATACCGGGTGCGGCATCCGTCCTTTCGCTGCCCGAG

ATGATGCGCCTGATGGTGTTTCAGGATTATGGTTTTTTGGCCGAAGTGATGTTTGTGCTG

ACTTTCGGC

>FA_1090_pDFB7

CGGGATCGACAATAAATCTGCCTTGGTGGTAAAGGATTTGGCTGTCGGAATAGGCGTTGT

CCACCTTGATGTCGACAGAGTTCTGACCCATGCCGCGCAGCGTCAGGAATTGGGACGTGC

CGTTGCCGCCGCCGAAATCGATGGAGGGCTCTTCTTTTAAGAGTTCGCGCATATCGGTTG

CGGTGCTTTCGTCTTTTTGTTGAAGCGTAACGATGTTGGTACGGATTTTGCTGCCTTGGC

GGTCGCCTTTTACGGTAACGGTATCCAATGCGACATTGGCATTATTTTCTGCCGCGTGGG

CAAAGCCGGCGGCAAGTGTGAGCGAGAGCAGGCTGAGGCGGAAAAACGGGGCGTTCATTT

GTTCGTCCTTTTGAGTGTATGAAGGGAAGTTAAGCCAAACCGTTAAGGTTTGGCAGGATA

AGAAAAAATAATAATAATTATTTTTGTTTATATTAGCGGGGGGGGGGGGGTTGTGTAAAG

CTGATTATCGTTTTTATTTGCGAAGCGTTGTTTTTTGTTGACAGGTTTTGTCGGAAATGT

ATAGTGGATTAACAAAAATCAGGACAAGGCGGCGAAGCCGCAGGCAGTACAGATAGTACG

GAACCGATTCACTTGGTGCTTCAGCACCTTAGAGAATCGTTCTCTTTGAGCTAAGGCGAG

GCAACGCCGTACCGGTTTAAATTTAGTCCACTATAAAAAACGGCGGAAATAAATTTTTTT

CCGCCTCACTTGAATTTTCCCGCACACACCCTAATTTTGCCAACTTGTACGGGCAGGCCG

TTAGGCGGCAGCCCGGTTTCCACTTCAATTTGTCCGAACCG

>FA_1090_pDFB8

GGCATACTCTTGGGCGGTTTGAATTTTATGAACATCGTAACCTACATCACTGCGCACCAA

ATGATGAAGGACAGTCCCACGCTGGTTTTTGCCGGTATGAATATCGGTGTGATTGTTTTA

GGGACGCTTTCGGGCGCATTGTTCTTTAAGGAAAAAATCAACACAATCAATACGGCAGGC

ATTGTACTGTCATTATGCTCCATTGCCTGCCTGTTCTATTGGACAGAAGTTAAGGCATTG

TTCGGCATATAATCCGGCTGATTGATATAACAAAATGCCGTCTGAAACAGGAAAATGCTG

CTTCAGACGGCATTTTGTTATACGGGCAGATTCAAAATTTAATTAATAATAGTTATTTTT

AATTATAATTTAAATATTAATAATAATTAAAAATTTCAAATAATTCTTATTATGAGTGTA

TCTTAATAAAATTCATTTTATATTCTTAATAAGGAATACATGATGTCCGGATTCTCCCCC

AAACCTAAGACCATTATCCTAAGCCTTGCAGGTGCATTTGGCGCATTGGCTTTTGCAGAC

ACCCCGAACAATACCGAACAGCAGAAAGAACTGAATACCATAGTCGTCCATGGCAAACGC

AGTGCCGACCAAAAAGGCGCGGACGACGTGTATTACAAAAACGTCTCCAACGCCTACGTC

GGCAAAGAATACCTCGAACGCTACCGCGTCCAATCCGCCGGCGACGTACTCAAAGGCCTA

AACGGCGTGTACAA

>FA_1090_pDFB9

GGCTTACCGGTTTTCTTTTTTAGTTTCCGCAGGCGTGCTGCCGAAATATTGCCTGAATGC

CTGAATAAAGCTGGAAACGTGGCGCTAGCCGCACAAATACGCGGTTTCGCCCACGTTTTT

GCCCCCGTTTTGCAACAGATAGAGCGCGTGCTGCATTTGTTTGTGGTGCAGCCACTCGCT

TGCCGTAATGCCGAAATGGTCGCGCATACGGCGTTGCAGCGTCCTTTCGCTGATGTTCAG

CGCGGCTGTCAGCCGGTTGACTTGGTGTGCGCCGCCGTCGAACGCGGCATTCAGGGTGCG

GCTGAAGTCTTCAGACGGCATAGCGTCTGCTTCCGCCGTTTGCCCCGCCGCCGGCTCGAT

GCCGTCTGAAACCGTGTCCCACAAATCCGACAGCAGCCGCAACACGTCCGCCTCGCGGCG

CAATGTTTCGCCCAAATGCCCCTTTGGGACGGTTTGCAGGCAGGATGCCGCCAAGCCGCG

CAGGTTTGGGGGCAAATCCCATATCCTGACCGGTTCGCGGTAAAGCAGGGGTGCGAAACG

CGCGTATTCCGGACGCGGCAGCCATTGTTCCATACCTTTAATGGTCATTTTGACCGTTTT

GCCGCCGCGATAAAGATAGCGGCTGAACAGGACTTCTTCCCCGACGGCAATTAGGACAAT

CTTGCCGCCGTCCGCATCGATGCGGAAGCGGCGGCGGTTGATGCCGAAGTCCAAACGCCC

TTCCAGCAACAGCACGAACGACACATAAGGTTCGGCGAGGCGGCTGCTGCAAAAATCGCA

CCGTGCCGTTACCGTACCGCCGTGGATGGAAATGCCGTTGGACAAAGTATCGAAACGGTA

GCTGCCCTCCACATAAGCCGCCGCCGCTTTTGACCAGATTGACGAACCGGCTGCTGTTCA

AATGTTCTGCTTTTTCCATAGTTTTCTGATAAAGGTTATCATTTGAAAGATAACATTTTT

CGCCACAGCAAACAATCCGCCGCCGACAAAGGCATTTTCACAACACTTGCCGTCCGTATG

CCGTCTGAAAATTGCCCGTCTTCCCGATAAGCCTTAAAATACACGCCGTCAAACCTACCG

TCCGCCCGTACATGAGCCCATCCCCCTTTATCGAAATGAAAGACGTCGCCTTCGCGTATG

GCGACCGCCCGATTCTGAACGACATCAATTTCAGCATTCCGCAAGGCAATTTTGCCGCCG

TGATGGGCGGTTCGGGCAGCGGCAAAACCACGCTGATGAGGCTGATTACAGGACAGATTC

GTCCGCAGTCCGGGCAGGTTTTGATTGAAGGACGGGATTTGGCGGGTTTTTCGGCTGACG
