# Supplementary material for: Iron-regulated small RNA expression as Neisseria gonorrhoeae FA 1090 transitions into stationary phase growth
Source: BMC Genomics. 2017 Apr 21;18:317. doi: 10.1186/s12864-017-3684-8 (PMC5399841; doi:10.1186/s12864-017-3684-8)
Supplement: Supplementary file 4 — Fe regulated Nrf sRNAs; primer extensions and Northern blot analysis. (DOCX 1051 kb) [file 12864_2017_3684_MOESM4_ESM.docx]

Additional_file_4_Figure_S2_Northern_primer_extension_Fe_regulated_sRNAs_FA_1090: NrfA_NrfB_NrfC_NrfD_NrfE_NrfF_NrfG_NrfH_NrfI_SAM riboswitch

**A. NrfA**

FA 1090 11980..12123

CTAAAACGGTTGTTGCCGACCGTTATGCCGG**^**TTTATGAGCAAAGTGTCAGAAATAAGGGGCGCGTTAATAAAAAACGTCGGCGTTAAGGGAAGGGGATCGAGAATTTGAGCCGTTGTTTCAAAATGCCGTCTGAAATGGTTTGAGATTCAGACGGCATTTAAACAGTTCTGCGCC

| →NGO_0007 | → NrfA cis to 3’ end of ←NGO_0010 | ←NGO_0011 |
| --- | --- | --- |

**B. NrfB**

FA 1090 186157..186314

CGGCAGATTTGCGCTG**TATAAT**GGTTTG**^**TTGGATGGCGGATAGTACGGAATCTCCCATGATTTTCCTTCTGTTTGTTTCTGTTTGTTCGGAATGATAGGCTAAACGGCTGCTCTCGGGCAATACGCCTGTTGCGTTTCGTTGGAAAATGCCGTCTGAGCGTTTCAGACGGCATTTGTGCTGTTGCA

| ←NGO_0184 | → NrfB cis to 3’ end of ←NGO_0185 | ←NGO_0186 |
| --- | --- | --- |

|  |
| --- |
| **C**. **NrfC**  311 bp  FA 1090 199898..199601 reverse strand  AGAAATTTTTAATATATTAATCAATAAATTAATTTTATAAAATAAAAATATTGTCAACAATCTTTTGACTTATCGCCCAAACCTCTG**TATATT**TTCCTACAGTA**^**AATTGTTGACAATCCATACGCCCGCATATGCGCCGCCTAAGGATAAATCCTCCCGCCGGACAACGGGGGCAAGGGATAGGATGCGATATTTCCATATTCAAACAAGGGATTTGTTTCACGCACAGGGCGGCACATCGGCAAAATCCCCGCGCCGGTCCGGCAGGGCTTGCGCCCATCCCGGACAAGCCCCGACCCCGCCTTTCCGAAAGACGGGCCCAACCATTAAGGAAACTTTAATCAAAATGAAAAAACACATATGGGCGGCATCTTCTATCGGCAGAACCTTTAAACTGGTGGCTATCGGCAGAACC 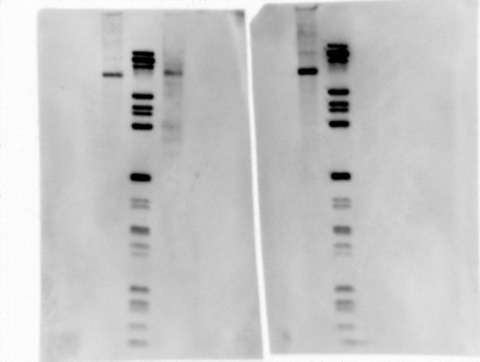 |

| ←NGO_0198 | ← NrfC trans | ←NGO_0199 |
| --- | --- | --- |

**D. NrfD**

FA 1090 275392..275583

AAT**AATAACTATAATTAT**CTTCGTCATAGT**^**CATTTTGTATACTATGTACCGAAAGGCTGTTCGGATTGGTAATTAAAGATTTACCCGTTAGTGTGATTGTGGAGGCGTGGTCTGTGTTGTGGTTGACAATGCGCGCGCCTTCATCCACGTTGCGGATGTGTTCAAAAGTCAAGTCATTGCCATTGGCATCCAAACGACCGCCACGGAAACCGAAATACAGAT


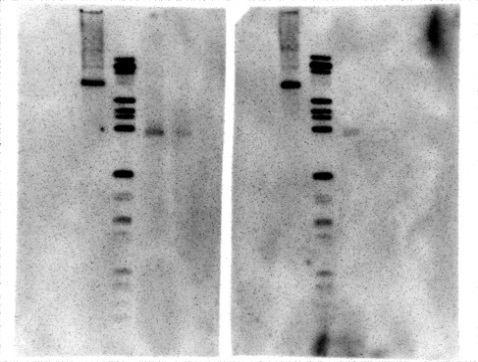


192 bp

| →NGO_0274 | → NrfD cis ←NGO_0275 | →NGO_0276 |
| --- | --- | --- |

163 bp


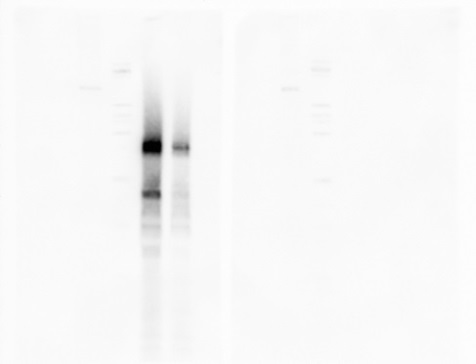


**E. NrfE**

FA 1090 637363..637525

CGTTTTGATT**TAGAAT**CTCCCC**^**AATATTTTTATCTTCGGACGGCATTTCATCATGCAAGGCAAAACGTTTACAACCGCTTACTGCTATTGGTACCACACAGACGTGCGGTGCTTTTTGCGCTTGCTTGAAATCCATCCCATACGCAAAACAGGCCGCTCGAAAACGGGGCGGCTTTTTTGTTACC

| ←NGO_0646 | → NrfE cis to 5’ end of ←NGO_03400 psuedogene | →NGO_0647 |
| --- | --- | --- |


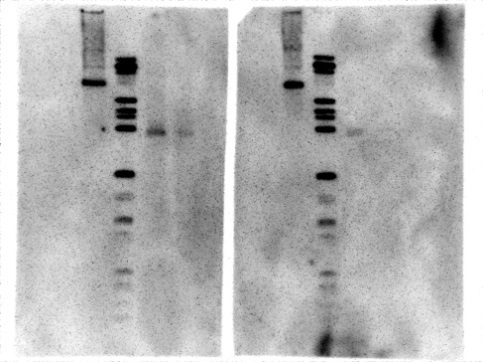


**F. NrfF**

FA 1090 771634..771825 reverse strand

AGGT**CTTTGACAT**TGTCTT**^**TCGGAATCAGGACGTGTTTGATGCCGCCGCGAAGCGCAGCCAACAGTTTTTCCTTCAGACCGCCGATCGGCAGGACTTCGCCGCGCAGGGTAATTTCGCCCGTCATGGCAACATCGGCACGCACCGGAATTTTGGTAAAGGCGGACACCATCGCCAAAGTCATAGCAATGCCTGCGCTCGGACCGTCTTTCG

192 bp

| →NGO_0773 | ← NrfF cis →NGO_0775 | →NGO_0777 |
| --- | --- | --- |

190 bp

**G. NrfG**

FA 1090 1308534..1308722

CAAAAAACAG**TGTAA[T**ATAGTGG^TTAACAAAAATCAGGACAAGGCGGCGAAGCCGCAGACAGTACAAATAGTACGGCAAGGCGAGGCAACGCCGTACCGGTTTAAATTTAATCCACTATA**]**CCACAAAGCGGGATAGGCAATGCTTAATACGGTCAAAAAAATATGTCCGAAAAAACCGGGTTTCATTTTGAATCCGCACAAACGTTTTCAGA


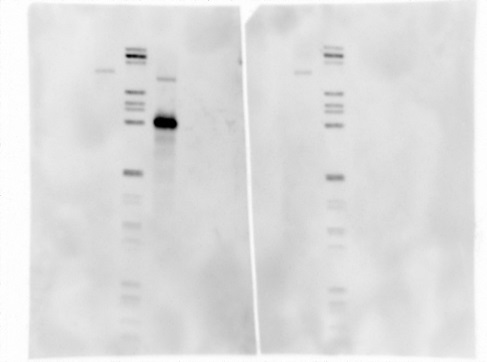


| ←NGO_1347 | →NrfG trans ←NGO_1349 |  |
| --- | --- | --- |

178 bp

**H. NrfH**

FA 1090 1458905..1459082

ATTTTTGTTAATCCAC**TATAAT**CAGGAAAGACAAAAAACC**^**TTCCGCCGTCATTCCCGCGCAGGCGGGAATCCGGCCCGTTGGGATTTTGCAACTTCAAATCAATCCGCAAACCGGAATCCCGTCATTCCCGCGCAGTCGTGAATCCGAACGCGTCCGCACGGAAATCCGCATCCCGTCATTCCCACGAAAGTGGGAATCTAGAATGTCAAATCTCAAG


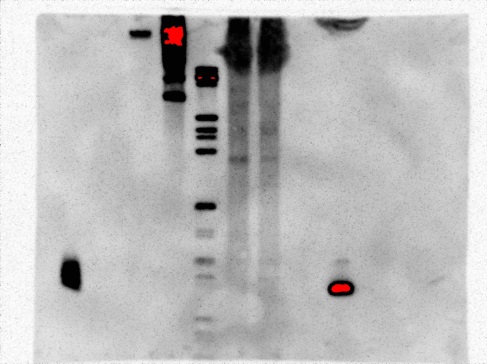


| →NGO_1494 | →NrfH trans ←NGO_07895 |  |
| --- | --- | --- |

**I. NrfI**

FA 1090 1996364..1996568

CTGTATTA**TATAAT**GGGGA**^**GAATATCTTAAATAACCAAGCATCCTTAATCATAATTTAGAGAAATTATTTTTTCAGAGTCTAACTCTTTTAATATGATTAACTTATTAACTTTATCTATTTCCCAGTCTGATATTATATCGGTAGATACTGAATTCTCTCTTATTAAATTTTTATCTGTAATAATTGCACCCAACTCATGAATAATACAAATTTTTTGATTATCCAATAAATAAATATCAAAAACTATTCCATCTATATTTAGTATGTTAACTTTTGAATTTTGTATAGAAA

| →NGO_2025 | → NrfI cis ←NGO_2026 | ←NGO_2027 |
| --- | --- | --- |

**J. SAM Riboswitch**

FA 1090 118200..118354 TCTGAAAAAGGGAAGTATTGCGGC**^**AATATGCCTTTTCTGCTACGATGCGCGCTGCATTAAGAGTTGGGAATTCCATGCCAACCTGCTTTTCAAACGGAAAGGTAAGGTGGACGGTTGAAAAACCGATGTGGCTCGCCGGAGCAATCCAAACCCGCTTGATGCGGGaaTTTTTTTGCCTGtacgaaacgtacggacagagattccaaagcgccgttta**aataggaatattt**ctcaactgaatggcacgaatagggaaattttgctatatttcccgctgtcgacattatgtcatacaacatgctgtctgaagaagatggtttgtttttcaaggaaaatttca*atg……


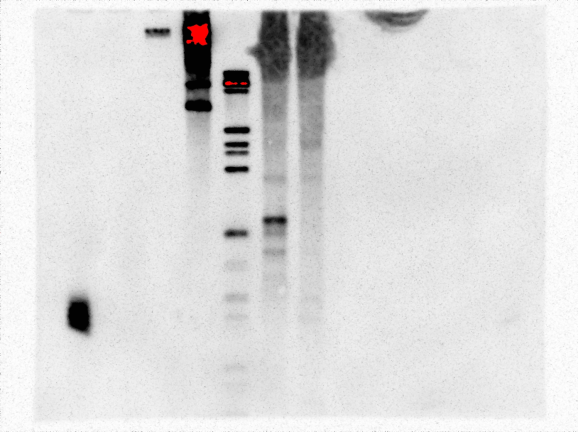


155 bp

|  | **Fe+ Fe-** |  |
| --- | --- | --- |

←NGO_0105 → SAM Riboswitch cis to 3’ end of ←NGO_00570 → **FB** NGO_0106

Red= Predicted Fur box

Blue= Rho independent terminator

**Bold type**= putative -10 promoter region

Underline= sRNA sequence complementary to cognate genes

**^** = Transcriptional start site determined by primer extensions or RNA-seq

**[ ]** = CREE sequence within brackets

* Translational start site for NGO_0106

5hr Fe+ total RNA was used for Northern blots except for SAM Riboswitch (3hr Fe+) and NrfD (3hr Fe-)
