# Supplementary material for: Iron-regulated small RNA expression as Neisseria gonorrhoeae FA 1090 transitions into stationary phase growth
Source: BMC Genomics. 2017 Apr 21;18:317. doi: 10.1186/s12864-017-3684-8 (PMC5399841; doi:10.1186/s12864-017-3684-8)
Supplement: Supplementary file 6 — Fe dependent NrrF regulated Nrs sRNAs; primer extensions and Northern blot analysis. (DOCX 611 kb) [file 12864_2017_3684_MOESM6_ESM.docx]

Additional_file_6_Figure_S3_Northern_primer_extension_Fe_dependent_NrrF_regulated_NrsA, NrsB ,NrsC, NrsD, NrsE

**A**. **NrsA**

FA 1090 327266..327508 reverse strand

TATAAAATCTACAATCATGCTTCCATCAACAGCAAAACATGATATGATTGCCAACA**^**ATGACATCTCACAATAAATTTTCTAATTTTTATTGAAAAAATCAATAAATTAAGAATTCTCCCAACCGACAAATAAAATAAAGAAAGGGTCAATATGCAACACCGTAGAAGATTGGCAATTTACCAGGCATCCAAACGTGCTTCCTTTACCGGCAGGTCATCAGCCCCACAAAAACGTAAAGAACGTTGATTTGAAAAAAATGCCGTCTGAAGTCCTGCTTCAGACGGCATTTTTTACCGTTC


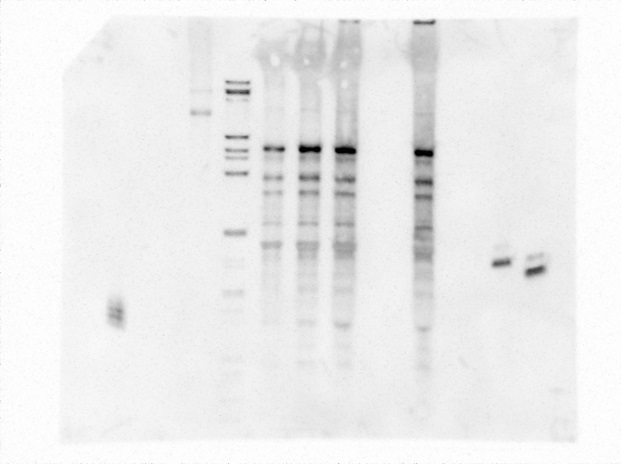


243 bp

**B. NrsB**

FA 1090 677432..677644

ATAATCACATAATGATAATTAATTTTTAACATCCTTGCTGTCCTATCATGA**TAAAAT**GACAATAGGGAT**^**GTTTTTCTGCTTTGGCTACGGCAAAACACCGTCGTCATTCCCGCGTAGGTAGGAATCCATATGCTTGGTTTCTCTTTTATTTCCAAACACTAATAAAACGGATAGGTCTGGATTTCCGCCTGCGCGGAAATGACGGAAATGTGCA

145 bp


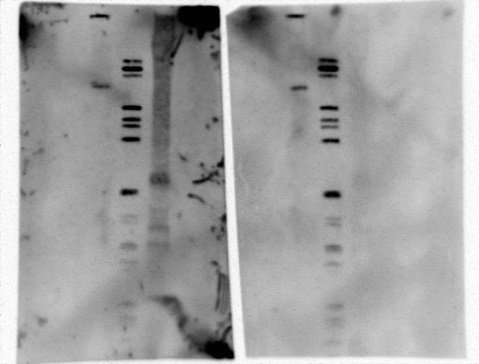


**C. NrsC**

FA 1090 1172443..1172616 reverse strand

ATTCGGACTGCACCTCCCGAATATATCTGCCTGCTGTTTCCTCTTTATTCAGCCTTTATAAT**^**ACTTGGACTTGTCGGGGTATTGTGCAGGCTTGATTCCGGATTGTCAACAATTTTCGGTCAAATTTTAAATGCCGCGTTTTAAAATGATGCCCGCCTGATTTTGCGGGCGGGCGGAAGGCGGGAATCCCGTTATTTTAAGCCATATATTAATTTATTGAATTAAAATAAATTTGTGCAATATAGTGGATTAACAAAAACCAGTACGGCATTACCTCGCCTTGCCGTACTATTTTTGTACTGTCTGCGGCTTCGT


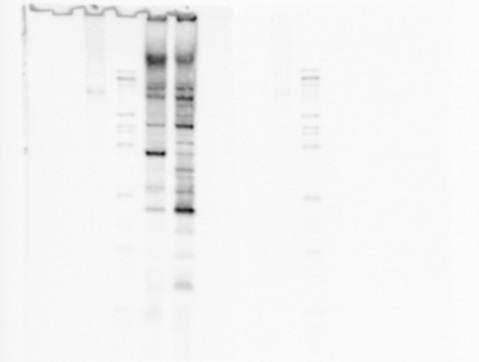


174 bp

**D. NrsD**

FA 1090 1608622..1608814

 AGGCGGTTAAAACAGCCGTTGCCAAGGCATTCAAACTGTAC**TAGAAT**AAAACCGTT**^**CCCTTAAAGGGGCTTGCAAGACTGTTCCGAAATATGGGCAGCCGCGCACGGGCGACAGGCGATGACAAGCCGTCCGTGCGTGTGATGGGGCGCGGAATGCGCCCCTTGTCGTATCTGCAAACGCCTACAAATCCCCAATCATCCCTTCAATAAAAATGCCGTCCGAACCTTCAGGCGGC**A**TTTTCCGTTTACC

193 bp


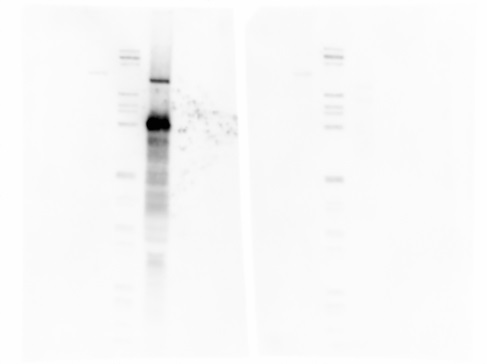


**E. NrsE**

FA 1090 1672491..1672668

AGGCGGGAATCTAGGTTTGTCCTCACGGAAACCGATATGCCGTCATTCCCGTAAA**^**AGCGGGAATCCGGTTCGTTCGGTTTCGTTTTTTTTTTTGAGTTTCGTGTAACTTCTGAATCGTCATTCCCGCGAAGGCGGGAATCCAGACCTTTAAACTCCGGCCATTCCCGATAAATTCCTGTTACTTTTCGTTGCTAGATTCCCGCCTGCGCGGGAATGACGAATGGCGGTGTAAA


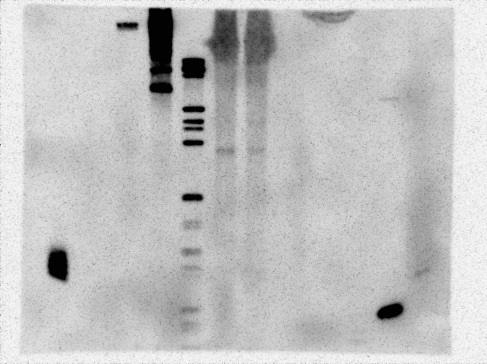


178 bp

Blue= Rho independent terminator

**Bold type**= putative -10 promoter region

**^** = Transcriptional start site determined by primer extension or RNA-seq

5hr Fe+ total RNA was used for Northern blots
