# Supplementary material for: Iron-regulated small RNA expression as Neisseria gonorrhoeae FA 1090 transitions into stationary phase growth
Source: BMC Genomics. 2017 Apr 21;18:317. doi: 10.1186/s12864-017-3684-8 (PMC5399841; doi:10.1186/s12864-017-3684-8)
Supplement: Supplementary file 7 — NrrF regulated Fe dependent Nrs sRNAs; prediction of secondary structure using RNAfold; IntaRNA predictions of potential RNA-RNA interactions between NrrF and the Nrs sRNAs. (DOCX 811 kb) [file 12864_2017_3684_MOESM7_ESM.docx]

Additional_file_7_Figure_S4_RNAfold_sRNA_secondary_structure_prediction_IntaRNA_target_prediction


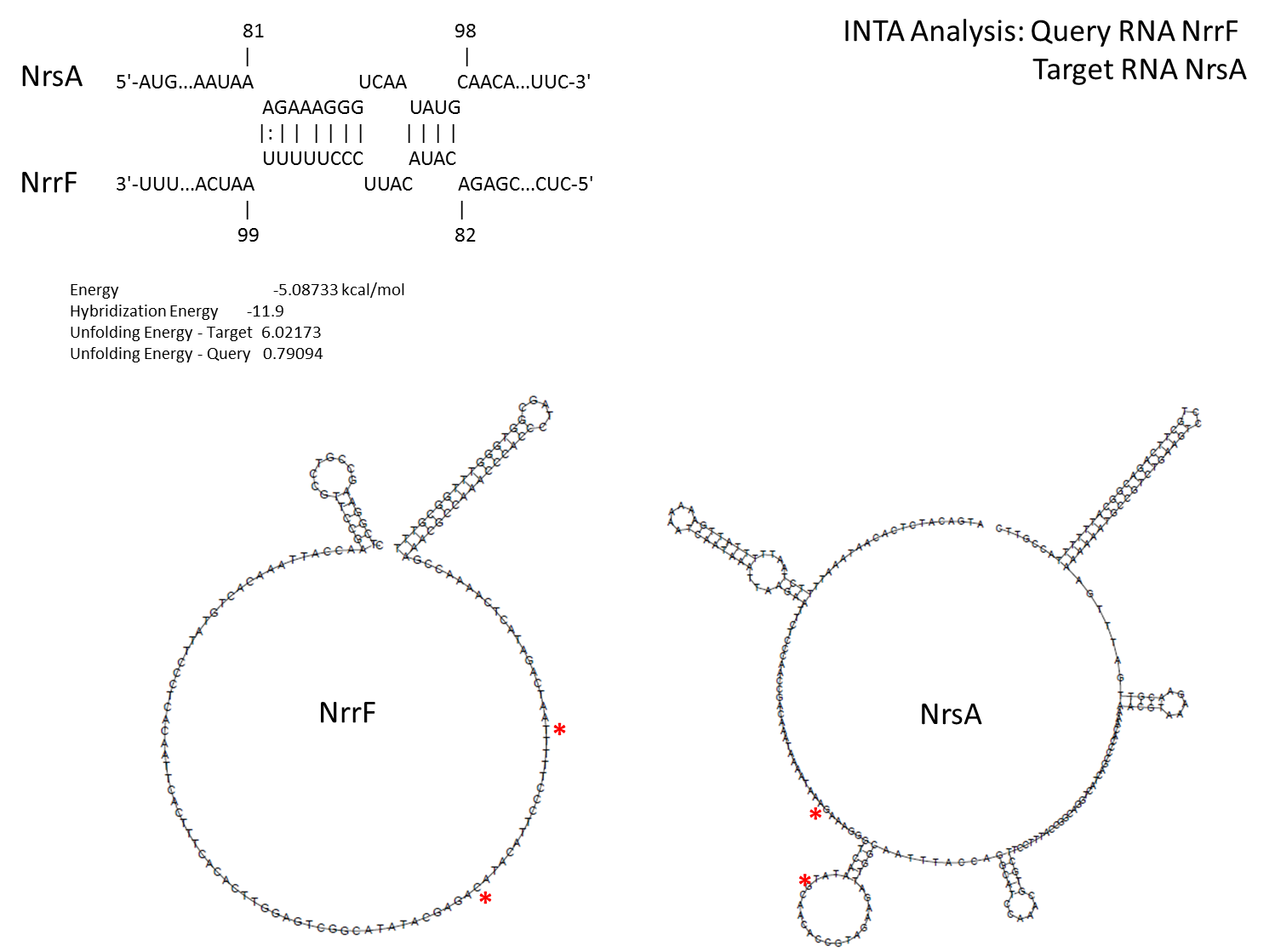


RNAfold secondary structure prediction

MFOLD prediction


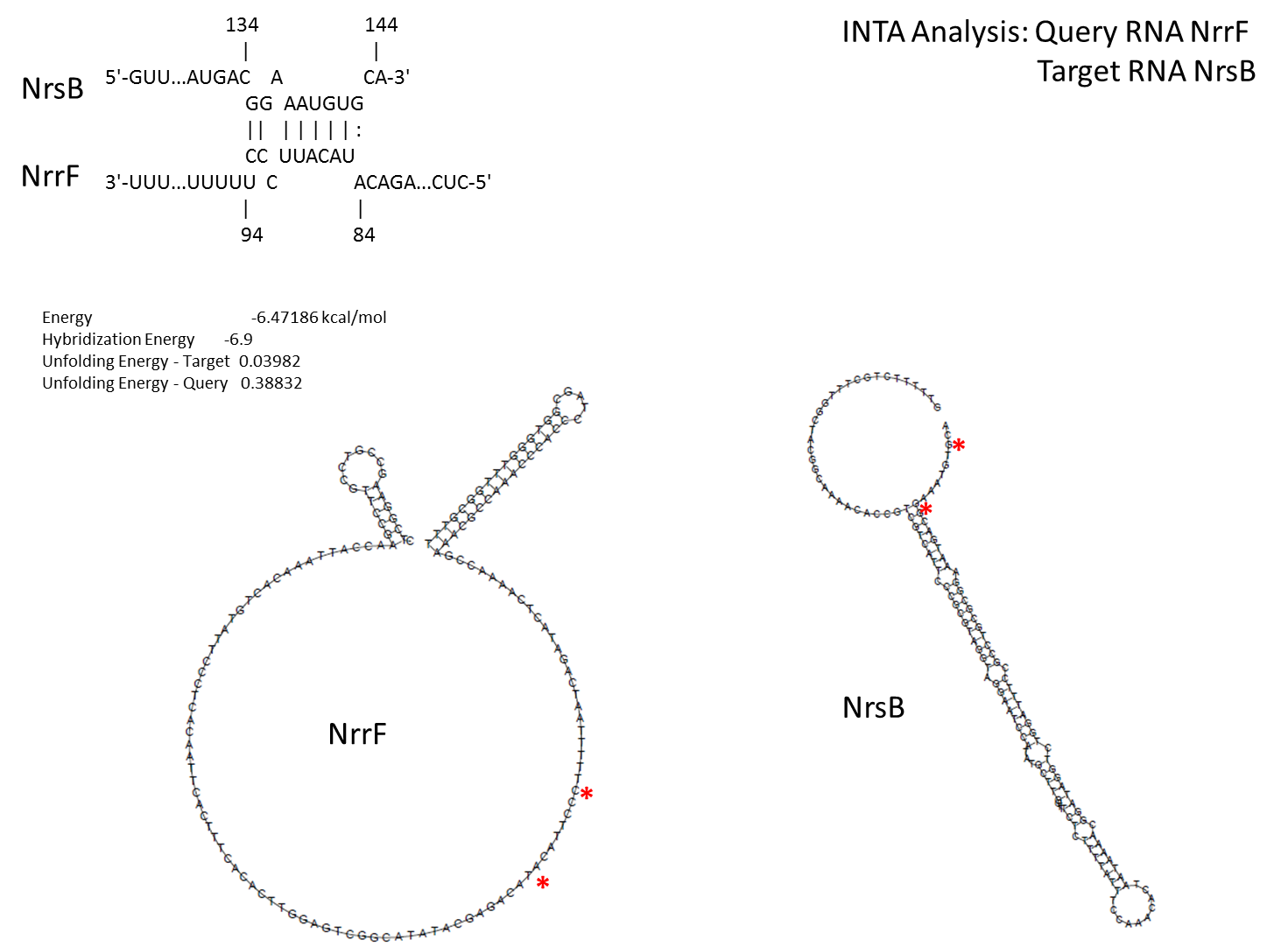


RNAfold secondary structure prediction


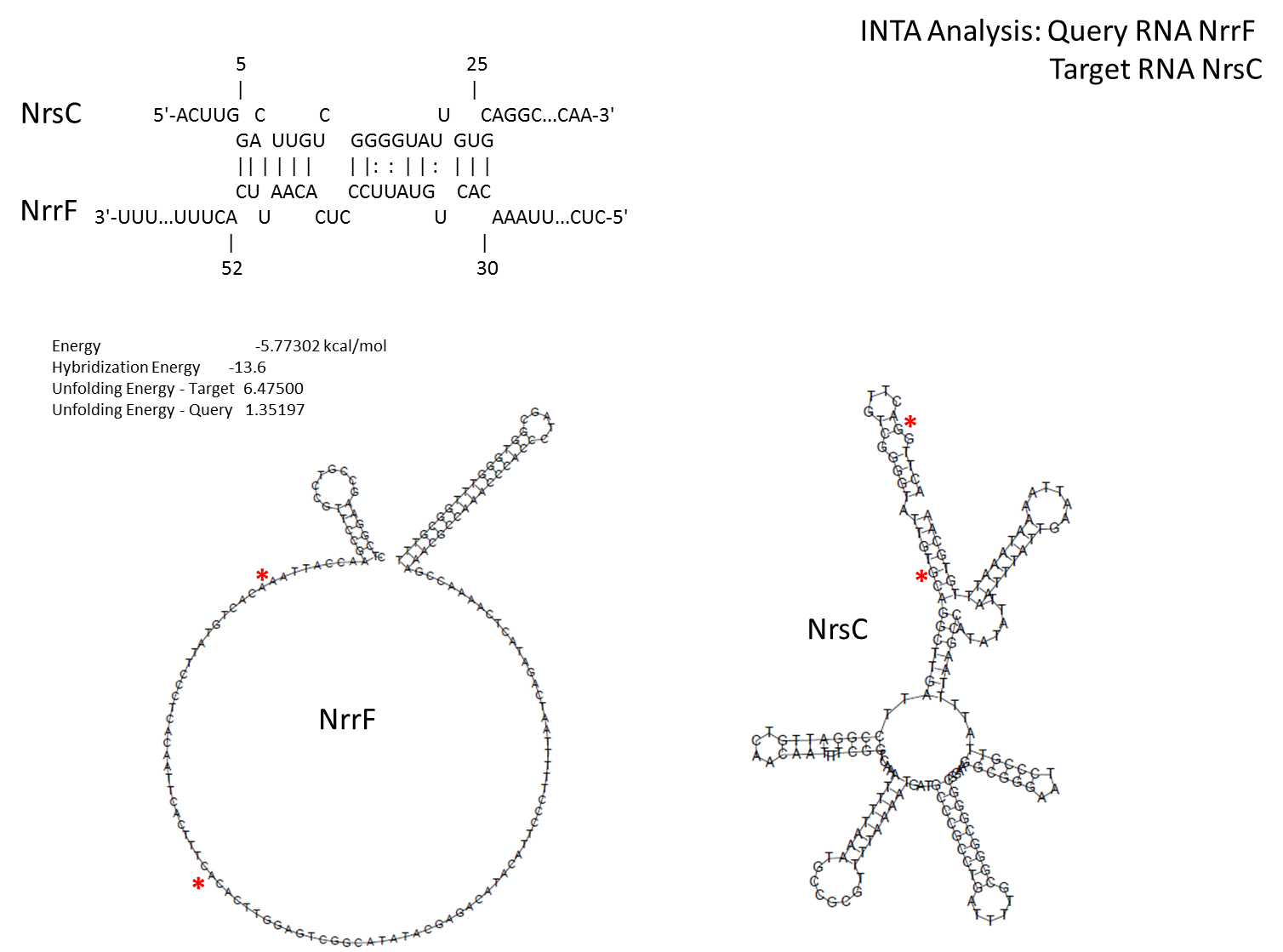


RNAfold secondary structure prediction


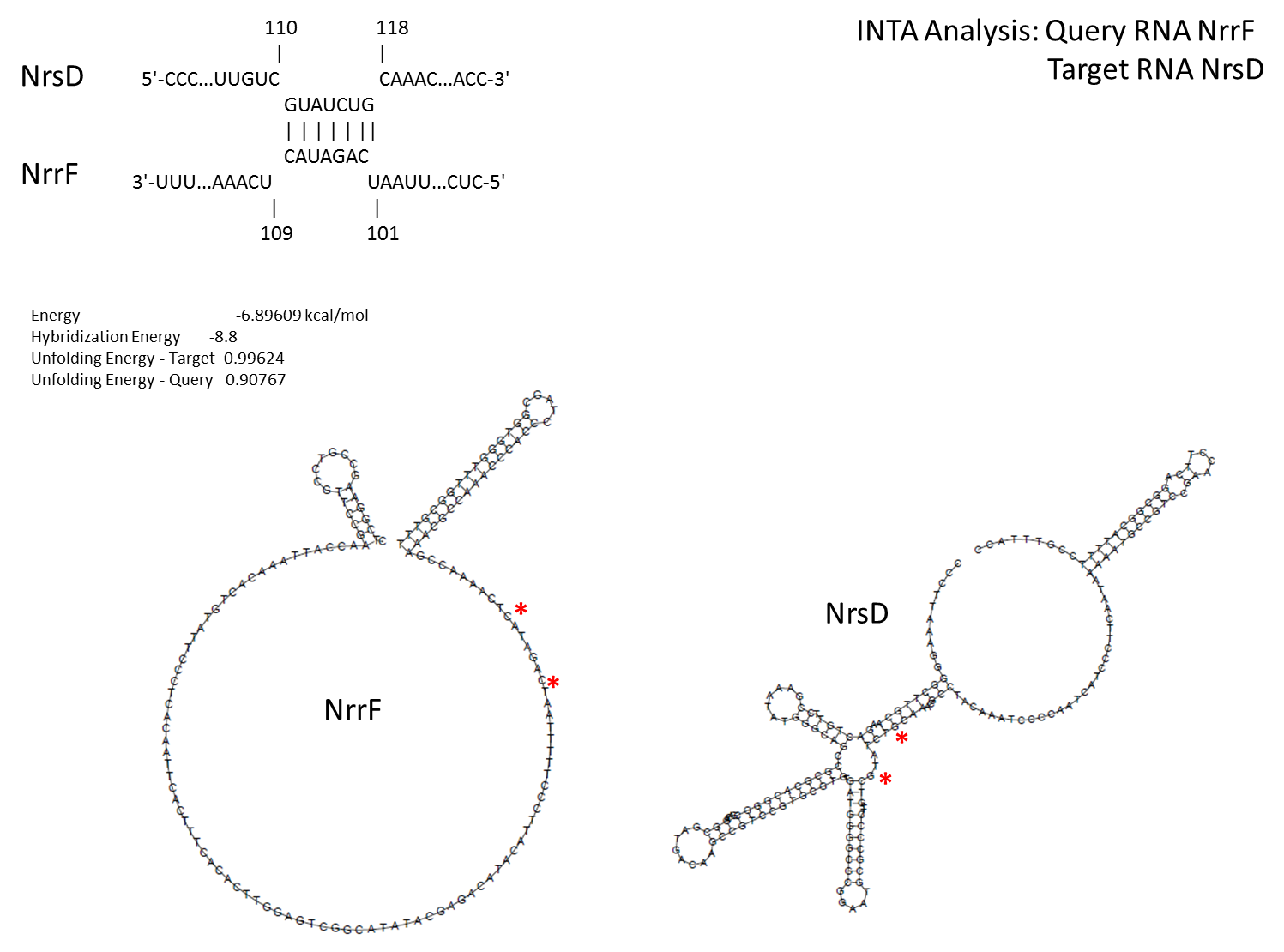


RNAfold secondary structure prediction


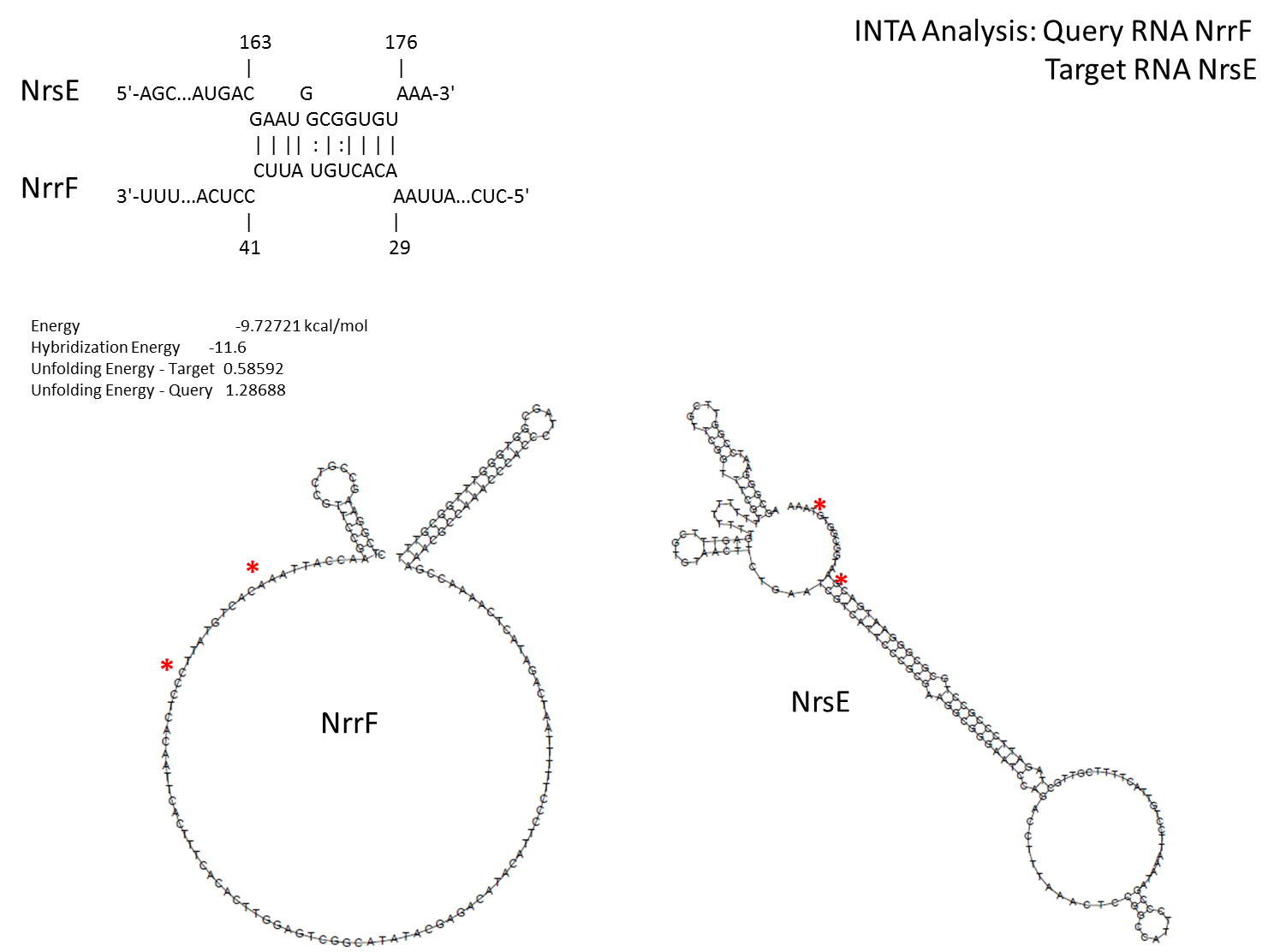


RNAfold secondary structure prediction
