# Supplementary figures and images for: Iron-regulated small RNA expression as Neisseria gonorrhoeae FA 1090 transitions into stationary phase growth
Source: BMC Genomics. 2017 Apr 21;18:317. doi: 10.1186/s12864-017-3684-8 (PMC5399841; doi:10.1186/s12864-017-3684-8)

## Conservation of Iron-regulated sRNA in Neisseriaceae

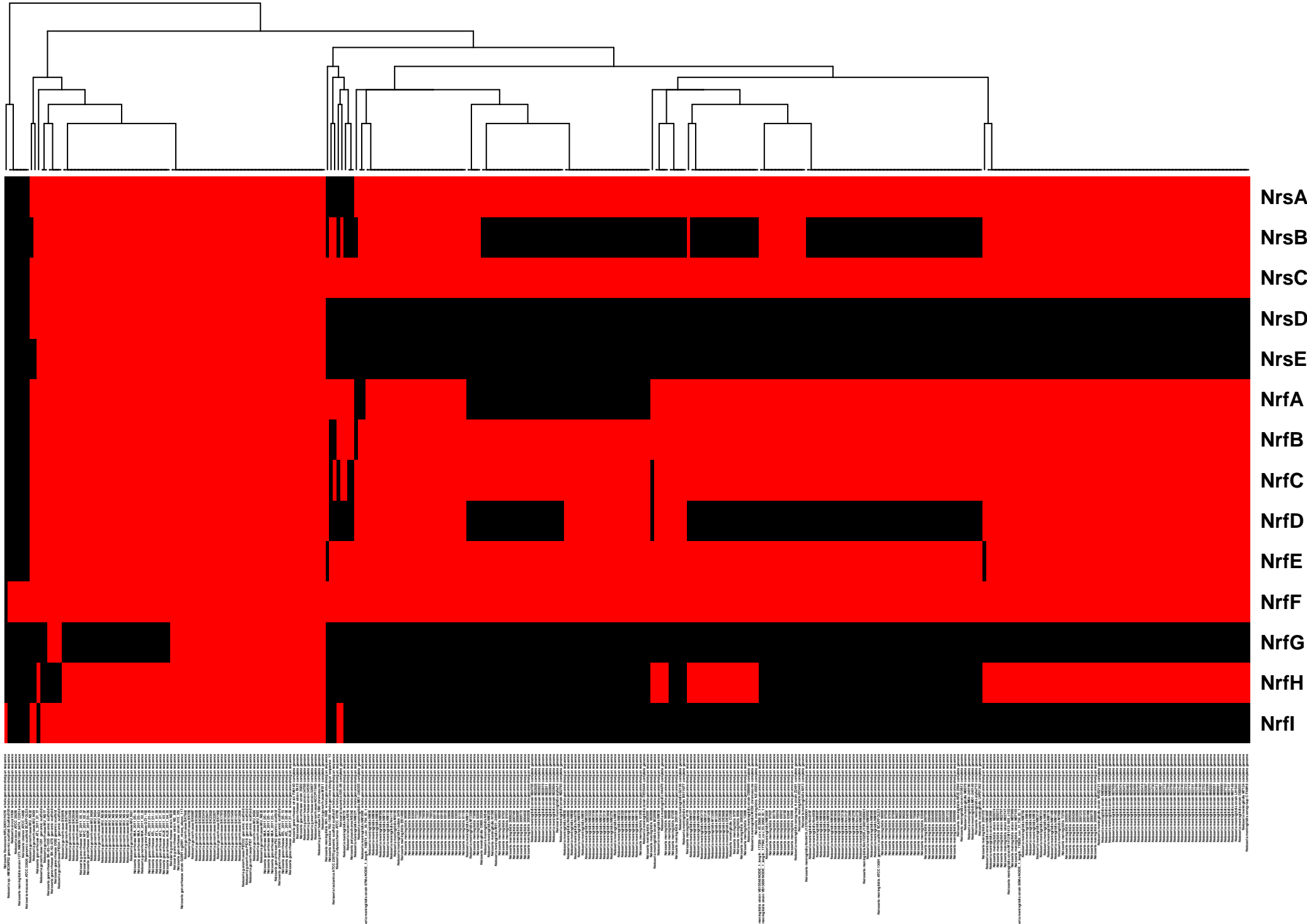

Supplement: Supplementary file 9 — Heat map cluster analysis of Fe regulated sRNA in the 345 Neisseria sp. genomes. Red indicates the presence of the sRNA and black designates that the sRNA is absent from that genome. (PDF 32 kb) [file 12864_2017_3684_MOESM9_ESM.pdf]
