# Supplementary material for: Iron-regulated small RNA expression as Neisseria gonorrhoeae FA 1090 transitions into stationary phase growth
Source: BMC Genomics. 2017 Apr 21;18:317. doi: 10.1186/s12864-017-3684-8 (PMC5399841; doi:10.1186/s12864-017-3684-8)
Supplement: Supplementary file 11 — List of the strains and the primers used in this study for real time PCR, primer extension, and Northern blot. (DOCX 18 kb) [file 12864_2017_3684_MOESM11_ESM.docx]

Additional_file_11_Table_S6_Primers_and_strains_used_in_this_study

| **Real Time PCR Primers** | | |
| --- | --- | --- |
|  | Forward primer 5’-3’ | Reverse primer 5’-3’ |
| NrfA | GGGCGCGTTAATAAAAAACG | AACAACGGCTCAAATTCTCGAT |
| NrfB | TGTTCGGAATGATAGGCTAAACG | TTTCCAACGAAACGCAACAG |
| NrfC | ATATGCGCCGCCTAAGGATA | CCCTGTGCGTGAAACAAAT |
| NrfD | GGCTGTTCGGATTGGTAATTAAAG | GCATTGTCAACCACAACACAGA |
| NrfE | TTACAACCGCTTACTGCTATTGGT | ATTTCAAGCAAGCGCAAAAAG |
| NrfF | TCGGAATCAGGACGTGTTTG | GGCGGTCTGAAGGAAAAACTG |
| NrfG | TACCACAAAGCGGGATAGGC | TTTTTGACCGTATTAAGCATTG |
| NrfH | CCGTTGGGATTTTGCAACTT | GGACGCGTTCGGATTCAC |
| NrfI | TCGGTAGATACTGAATTCTCTCTTATT | TTATTCATGAGTTGGGTGCAA |
| SAM Riboswitch | GGTAAGGTGGACGGTTGAAAAA | CAAGCGGGTTTGGATTGCT |
| NrsA | AAGAATTCTCCCAACCGACAAA | GGATGCCTGTAAATTGCCAATC |
| NrsB | GGCAAAACACCGTCGTCAT | TTGGAAATAAAAGAGAAACCAAGCAT |
| NrsC | GCAGGCTTGATTCCGGATT | CAGGCGGGCATCATTTTAAA |
| NrsD | TCGTATCTGCAAACGCCTACA | GTTCGGACGGCATTTTTATTG |
| NrsE | GGCGGGAATCCAGACCTTTA | TCTAGCAACGAAAAGTAACAGGAATTT |
| Por1 | TGTCCGTACGCTACGATTCTCC | AGCCAACGTGGTAAGATTCGC |
| NrrF | TCCGTTCCGAACCATTAAA | GCGTTTCGGTTTTGAGTATCT |

| **Primer Extension/Northern Blot** | | |
| --- | --- | --- |
|  | Primer 5’-3’ | Tm |
| NrfA | 6FAM TTCCCTTAACGCCGACGTTTTTTATTA | 57.1 |
| NrfB | 6FAM TTAGCCTATCATTCCGAACAAACAGAAACAA | 58.5 |
| NrfC | 6FAM ACGAATGAAATATTCGGCAATGATGCTGTTT | 59.4 |
| NrfD | 6FAM GCATTGTCAACCACAACACAGACCACGCCT | 65.8 |
| NrfE | 6FAM AGCGCAAAAAGCACCGCACGTCTGTGTGGTACCAATA | 68.6 |
| NrfF | 6FAM AAGGAAAAACTGTTGGCTGCGCTTCGCGGC | 68 |
| NrfG | FAM6 ACATATTTTTTTGACCGTATTAAG | 47.9 |
| NrfH | 6FAM ATGACGGGATTCCGGTTTGCGGATTGAT | 63.9 |
| NrfI | 6FAM TGGGAAATAGATAAAGTTAATAAGTTAATCA | 51.6 |
| SAM Riboswitch | 6FAM AAGCGGGTTTGGATTGCTCCGGCGAGCCAC | 70.4 |
| NrsA | 6FAM ATGCCTGGTAAATTGCCAATCTTCTACGGT | 61.7 |
| NrsB | 6FAM TTGGAAATAAAAGAGAAACCAAGCATATGG | 56.2 |
| NrsC | 6FAM AAAATAACGGGATTCCCGCCTTCCGCCCGCCCGCAA | 72.8 |
| NrsD | 6FAM TCGCCTGTCGCCCGTGCGCGGCTGCCCATA | 75.7 |
| NrsE | 6FAM TCTAGCAACGAAAAGTAACAGGAATTTAT | 55 |
| NrrF | 6FAM GCGTTTCGGTTTTGAGTATCT | 53.3 |

| **Gene Specific Primers for RT** | | |
| --- | --- | --- |
| sRNA | Primer 5’-3’ | Tm |
| NrfD | GCATTGTCAACCACAACACAGA | 56.2 |
| NrfE | ATTTCAAGCAAGCGCAAAAAG | 53.2 |
| NrfF | GGCGGTCTGAAGGAAAAACTG | 56.5 |
| NrfI | TTATTCATGAGTTGGGTGCAA | 52.4 |
| SAM Riboswitch | CAAGCGGGTTTGGATTGCT | 52.6 |

| Strain | Genotype | Reference |
| --- | --- | --- |
| FA_1090 | Wild Type | [J Bacteriol.](https://www.ncbi.nlm.nih.gov/pubmed/24039262) 2013 Nov;195(22):5166-73.  doi: 10.1128/JB.00839-13. Epub 2013 Sep 13. |
| LJ001 | *nrrf* mutant | [J Bacteriol.](https://www.ncbi.nlm.nih.gov/pubmed/24039262) 2013 Nov;195(22):5166-73.  doi: 10.1128/JB.00839-13. Epub 2013 Sep 13. |
| LJ002 | NrrF complement | [J Bacteriol.](https://www.ncbi.nlm.nih.gov/pubmed/24039262) 2013 Nov;195(22):5166-73.  doi: 10.1128/JB.00839-13. Epub 2013 Sep 13. |
